# Functional Disruption of a Chloroplast Pseudouridine Synthase Desensitizes Arabidopsis Plants to Phosphate Starvation

**DOI:** 10.3389/fpls.2017.01421

**Published:** 2017-08-15

**Authors:** Shan Lu, Chenyi Li, Ye Zhang, Zai Zheng, Dong Liu

**Affiliations:** MOE Key Laboratory of Bioinformatics, School of Life Sciences, Tsinghua University Beijing, China

**Keywords:** phosphate starvation, plant Pi sensitivity, DPS1, pseudouridine synthase, chloroplast translation, Pi demand

## Abstract

Phosphate (Pi) deficiency is a common nutritional stress of plants in both agricultural and natural ecosystems. Plants respond to Pi starvation in the environment by triggering a suite of biochemical, physiological, and developmental changes that increase survival and growth. The key factors that determine plant sensitivity to Pi starvation, however, are unclear. In this research, we identified an Arabidopsis mutant, *dps1*, with greatly reduced sensitivity to Pi starvation. The *dps1* phenotypes are caused by a mutation in the previously characterized *SVR1* (*SUPPRESSION OF VARIAGATION 1*) gene, which encodes a chloroplast-localized pseudouridine synthase. The mutation of SVR1 results in defects in chloroplast rRNA biogenesis, which subsequently reduces chloroplast translation. Another mutant, *rps5*, which contains a mutation in the chloroplast ribosomal protein RPS5 and has reduced chloroplast translation, also displayed decreased sensitivity to Pi starvation. Furthermore, wild type plants treated with lincomycin, a chemical inhibitor of chloroplast translation, showed similar growth phenotypes and Pi starvation responses as *dps1* and *rps5*. These results suggest that impaired chloroplast translation desensitizes plants to Pi starvation. Combined with previously published results showing that enhanced leaf photosynthesis augments plant responses to Pi starvation, we propose that the decrease in responses to Pi starvation in *dps1, rps5*, and lincomycin-treated plants is due to their reduced demand for Pi input from the environment.

## Introduction

Phosphorus (P) is one of essential macronutrients for plant growth and development. Inorganic phosphate (Pi), the major form of P that plants assimilate, however, is highly immobile in most soils due to its conversion to organophosphates by microorganisms or to its chelation with metals. As a consequence, P is one of the least available nutrients for plant uptake (Hinsinger, 2001), and Pi deficiency is a common nutritional stress that causes enormous losses in agriculture (López-Arredondo et al., 2014).

As sessile organisms, plants have developed elaborate strategies to cope with Pi starvation, including the remodeling of root system architecture (RSA); the increase of activities of high affinity Pi transporters; the induction and secretion of acid phosphatases (APases), ribonuclease, and organic acids; and the accumulation of starch and anthocyanins (Vance et al., 2003). These Pi-starvation responses are controlled by an elaborate gene regulatory network through both local and systemic signaling (Chiou and Lin, 2011). Many studies have indicated that transcriptional regulation plays a crucial role in controlling these responses (Jain et al., 2012). In *Arabidopsis thaliana* (Arabidopsis), PHR1 and its close homologs PHL1 (PHR1-like 1), PHL2, and PHL3, which belong to a MYB transcription factor family, are the central regulators of plant transcriptional response to Pi starvation (Rubio et al., 2001; Bustos et al., 2010; Sun et al., 2016). The activity of PHR1 is further regulated by SPX1, which forms complex with PHR1 to block its binding to the promoters of many Pi starvation-induced (PSI) genes (Puga et al., 2014). miR399, *PHO2*, and two other non-coding RNAs, *IPS1* and *At4*, form another signaling pathway that regulates Pi homeostasis in plants (Liu et al., 2014). Under Pi sufficiency, *PHO2*, which encodes a ubiquitin E2 conjugase, mediates degradation of several Pi transporters, thus preventing the overaccumulation of Pi in leaves. Under Pi starvation conditions, the induced miR399 binds to *PHO2* mRNA and mediates its cleavage, thereby stabilizing the Pi transporters located on the root surface. At the same time, the induced *IPS1* and *At4* begin to pair with miR399 to inhibit its silencing of *PHO2* mRNA and to therefore maintain Pi homeostasis in Pi-starved plants (Franco-Zorrilla et al., 2007).

In addition to the Pi responses mentioned above, a decrease in photosynthesis is another major physiological response of plants to Pi starvation (Jacob and Lawlor, 1992; Rao and Terry, 1995). Photosynthesis uses a large amount of Pi for synthesis of ATP and phosphorylated sugar intermediates. The decrease in photosynthesis under Pi deficiency is thought to save Pi for metabolic reactions, which are more important for plant survival. Meanwhile, most photosynthates are converted into starch in chloroplasts under Pi deficiency, which liberates additional Pi. In Pi-starved plants, the expression of a large number of photosynthetic genes is downregulated (Wu et al., 2003; Misson et al., 2005; O'Rourke et al., 2013). The suppression of photosynthetic gene expression even occurs in roots, a non-photosynthetic organ (Li et al., 2010; Kang et al., 2014). Such suppression is required for sustained root growth under Pi deficiency (Kang et al., 2014). Therefore, the reduction of photosynthesis under Pi starvation seems not to be a simple passive result of nutrient deficiency but rather an active cellular response that helps plants adapt to the stress. These results also suggest that chloroplasts might play an important role in regulating plant growth and development under Pi deficiency.

Chloroplasts of most vascular plants have a genome with about 120 genes and the machinery to express them (Wicke et al., 2011). Proteins produced by both chloroplast and nuclear genes are necessary for the biogenesis and function of the photosynthetic apparatus. Maintenance of the integrity of the photosynthetic apparatus is critical for its functions. Chloroplasts have also been reported to play an important role in plant development (Tiller and Bock, 2014), as well as in the responses to abiotic stresses, such as drought (Zhu, 2016). Although many individual components involved in Pi signaling have been identified, how the status of chloroplast affects plant sensitivity to Pi starvation has not been well explored.

The chloroplast has been recently proposed to sense environmental stress and to communicate the presence of stress to the nucleus via retrograde signals (Fernández and Strand, 2008; Zhu, 2016). For example, several reports have indicated that the defects in chloroplast protein translation affect plant responses to heat (Yu et al., 2012) and cold stresses (Rogalski et al., 2008; Zhang et al., 2016) by impairing the integrity of the photosynthetic apparatus. Here, we show that a mutation in a chloroplast-localized pseudouridine (Ψ) synthase, SVR1, reduces the magnitude of plant responses to Pi starvation. SVR1 is required for proper chloroplast rRNA processing and protein translation (Yu et al., 2008). Furthermore, we provide pharmacological evidence that inhibition of chloroplast translation reduces plant sensitivity to Pi starvation. Based on the current and previously published findings, we propose that chloroplast translation, which may affect plant demand for Pi input from the environment, is an important determinant of plant sensitivity to Pi starvation.

## Materials and methods

### Plant materials and growth conditions

All *Arabidopsis thaliana* plants used in this study were of the Columbia ecotype unless indicated otherwise. The *svr1-2* T-DNA insertion mutant was kindly provided by Dr. Fei Yu (Northwest A&F University, China). The *rps5* point mutation mutant was a kind gift from Dr. Li Li (Cornell University, USA).

The standard Pi-sufficient (P+) medium was one half-strength MS medium (Murashige and Skoog, 1962). The components of the MS medium include: 20.62 mM NH_4_NO_3_, 18.79 mM KNO_3_, 2.99 mM CaCl_2_, 1.5 mM MgSO_4_, 1.25 mM KH_2_PO_4_, 5 μM KI, 100 μM H_3_BO_3_, 100 μM MnSO_4_, 30 μM ZnSO_4_, 0.1 μM Na_2_MoO_4_, 0.1μM CoCl_2_, 0.1 μM CuSO_4_, 100 μM Na_2_EDTA, 100 μM FeSO_4_, 27 μM Glycine, 0.55 mM myo-inositol, 4 μM nicotinic acid, 2 μM pyridoxine•HCl, 0.2 μM thiamine•HCl, 5.1 mM MES, 1% (w/v) sucrose and 1.2% (w/v) agar (Sigma catalog no. A1296). The pH of the medium was adjusted to 5.8. In the Pi-deficient (P−) medium, 1.25 mM KH_2_PO_4_ was replaced with 0.625 mM K_2_SO_4_. The seeds were surface sterilized with 20% (v/v) bleach for 12 min and were then washed four times with sterile-distilled water. The seeds were subsequently sown on Petri plates containing P+ or P− medium. The agar plates were kept at 4°C in the dark for 2 days and then were placed in a growth room with a 16-h-light/8-h-dark photoperiod at 22–24°C. The light intensity was 100 μmol m ^−2^s ^−1^.

### Mutant isolation

The Arabidopsis T-DNA activation tagging library was generated with the transgenic plants carrying a luciferase (LUC) reporter gene under the control of the *AtPT2* promoter (*AtPT2::LUC*) (Karthikeyan et al., 2002; Koiwa et al., 2006). Seeds of the T_2_ progeny of the original T-DNA lines were germinated on P− medium and grown for 7 days. A luciferin solution (100 mM) dissolved in 0.1% (v/v) Triton X-100 was sprayed uniformly on the surface of the seedlings. Luminescence images were taken by exposing the plants to an Andor iXon CCD (charge-coupled device) camera (Andor Technology Ltd., South Windsor, CT, USA) for 5 min. Putative mutants with altered luminescence relative to the wild type (WT) were selected, and their mutant phenotypes were reexamined in the next generation.

### Quantitative real-time PCR analysis (qPCR)

qPCR analyses were performed essentially as described in Wang et al. (2011). The genes and the primers used in qPCR analyses are listed in Supplementary Table 1.

### Analysis of APase activity

The APases on the root surface of Arabidopsis seedlings were stained as described by Lloyd et al. (2001). An agar (0.5%, w/v) solution containing 0.01% (w/v) 5-bromo-4-chloro-3-indolyl-phosphate (BCIP) was evenly overlaid on the root surface. After 8 h of color development at 23°C, the seedlings were photographed with a camera attached to a stereomicroscope (Olympus SZ61).

### Analysis of anthocyanin accumulation

For the measurement of anthocyanin, shoots of 12-d-old seedlings grown on P+ or P− medium (0.6% agar) were extracted overnight with extraction buffer (18% C_3_H_7_OH [v/v], 1% HCl [v/v]). After centrifugation for 5 min at 13,000 rpm, the upper aqueous phase was subjected to spectrophotometric quantification at 530 and 650 nm. Relative anthocyanin levels were expressed as (A_530_-A_650_)/g fresh weight. For observation of anthocyanin accumulation in leaves, the shoots of the 12-days-old seedlings were photographed with a camera attached to a stereomicroscope (Olympus SZ61).

### Analysis of starch accumulation

For starch analysis, 10-days-old seedlings were immersed in 95% (v/v) ethanol until the pigments were removed and were then washed twice with distilled-deionized water. The seedlings were then stained with 5% Lugol's solution (5% I_2_[w/v], 10% KI[w/v]) for 10 min. After one wash in distilled-deionized water, the samples were kept in distilled-deionized water until the background became clear, and starch accumulation was documented with a camera attached to a stereomicroscope (Olympus SZ61).

### Quantitative analysis of cellular Pi and total P contents

Cellular Pi and total P content were measured as previously described (Wang et al., 2011).

### Genetic identification of the *DPS1* gene

A positional cloning approach was first used to roughly map the chromosomal location of the molecular lesion in *dps1*. The mapping population was generated by crossing *dps1* to a plant of the *Landsberg erecta* ecotype. The F_2_ progeny that displayed the *dps1* phenotype were selected, and DNAs from these seedlings were extracted for molecular mapping. The sequences and chromosomal positions of the molecular markers are listed in Supplementary Table 2. After rough mapping, a whole-genome sequencing approach was used to find the putative mutation in *dps1*. DNA sequencing and data analysis were conducted at the Purdue University Sequencing Facility.

### Genetic complementation of *dps1*

For genetic complementation of the *dps1* mutant, the WT genomic sequence of the *DPS1* gene was amplified from the Arabidopsis genomic DNA by PCR. The PCR product was ligated to the pZhou vector between the 35S CaMV promoter and the NOS terminator. The resulting construct was named *35S CaMV::DPS1*. This construct was transferred into *Agrobacterium tumefaciens* strain GV3101 and into *dps1* plants by the floral dip method (Clough and Bent, 1998). The transgenic plants were selected on hygromycin-containing 1/2 MS medium.

### Protein extraction and immunoblotting analysis

The seedlings were ground to fine powders in liquid nitrogen. One volume of ice-cold extraction buffer (0.1 M K-acetate, 20 mM CaCl_2_, 20% glycerol [v/v], 2 mM EDTA, 0.1 mM phenylmethylsulfonyl fluoride, and pH 5.4) was added to the powders. Samples were gently mixed and incubated on ice for 30 min and were then centrifuged at 13,000 rpm at 4°C for 16 min. The supernatant was transferred to a new ice-cold tube, and the centrifugation was repeated twice. About 10 μg of denatured protein was separated on 10% SDS-polyacrylamide gels. After electrophoresis, the proteins were transferred to a polyvinylidene difluoride membrane in buffer (25 mM Tris, 43 mM glycine, 10% methanol [v/v]) and subjected to western blot analysis using a monoclonal antibody against Arabidopsis RbcL protein.

## Results

### Identification of the *dps1* mutant

**Figure 1:**
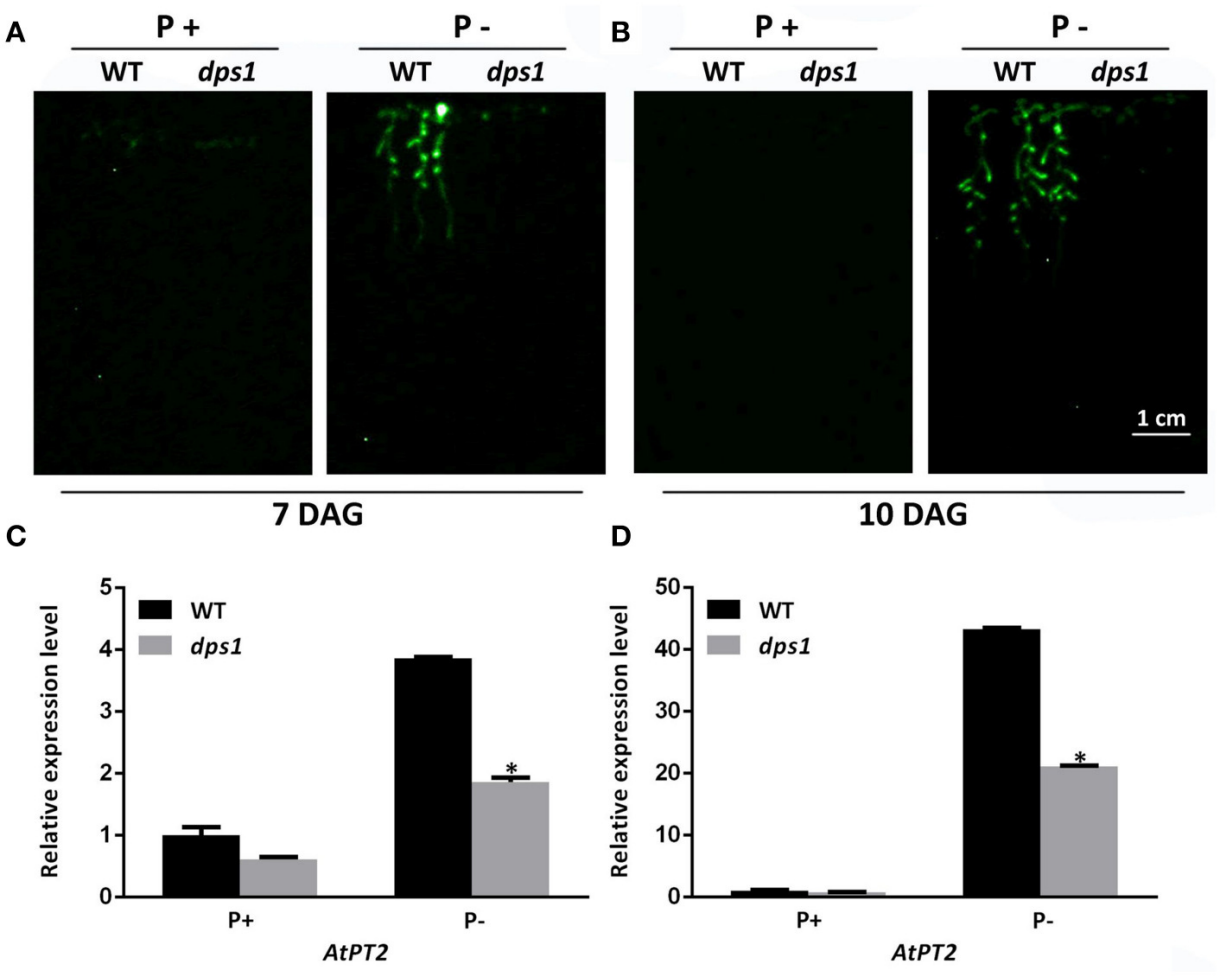
Analysis of *AtPT2::LUC* expression in WT and *dps1* seedlings. **(A,B)** The expression of *AtPT2::LUC* in 7-days-old **(A)** and 10-days-old **(B)** WT and *dps1* seedlings grown on P+ and P− media as indicated by luminescence signals. **(C,D)** Relative expression of the endogenous *AtPT2* gene in 7-days-old **(C)** and 10-days-old **(D)** WT and *dps1* seedlings grown on P+ and P− media. In **(C,D)**, three independent experiments were performed with similar results and one representative experiment is shown. Values are means ± SD of three technical replicates and represent fold changes normalized to transcript levels of the WT on P+ medium. An asterisk indicates a significant difference relative to the WT (*t*-test, *P* < 0.05).

To identify novel key factors that determine plant sensitivity to Pi starvation, we screened an Arabidopsis T-DNA insertion library for mutants with altered responses to Pi starvation. This library was constructed by transforming a transgenic line carrying an *AtPT2::LUC* (luciferase) reporter gene (herein referred as the wild type [WT]) (Koiwa et al., 2006). The *AtPT2* gene (also named *AtPht1;4*) encodes a high-affinity Pi transporter. The transcription of *AtPT2* is specifically induced by Pi starvation, especially in roots (Karthikeyan et al., 2002). As a result, the expression level of *AtPT2* could be estimated by examining the level of luminescence signal after luciferin, a substrate for luciferase, was sprayed on the surface of plants. For the mutant screen, the seeds from the T-DNA insertion library were directly sown on a Pi-deficient (P−) medium. Seven days after germination (DAG), expression of the *AtPT2::LUC* gene in the seedlings, as indicated by luminescence, was recorded with a CCD camera. One mutant, designated *dps1* (d*ecreased sensitivity to* P*i* s*tarvation*1), was identified. The induction of luminescence was almost undetectable in this mutant when it was grown on P− medium for 7 or 10 days (Figures 1A,B). To ensure that the impaired induction of the *LUC* gene was not due to a mutation in the *AtPT2::LUC* transgene, we analyzed the expression level of the endogenous *AtPT2* gene by qPCR (Figures 1C,D). The results showed that the expression of *AtPT2* was greatly reduced, indicating that *DPS1* plays an important role in regulating *AtPT2* expression.

### The *DPS1* mutation reduces PSI gene expression

**Figure 2:**
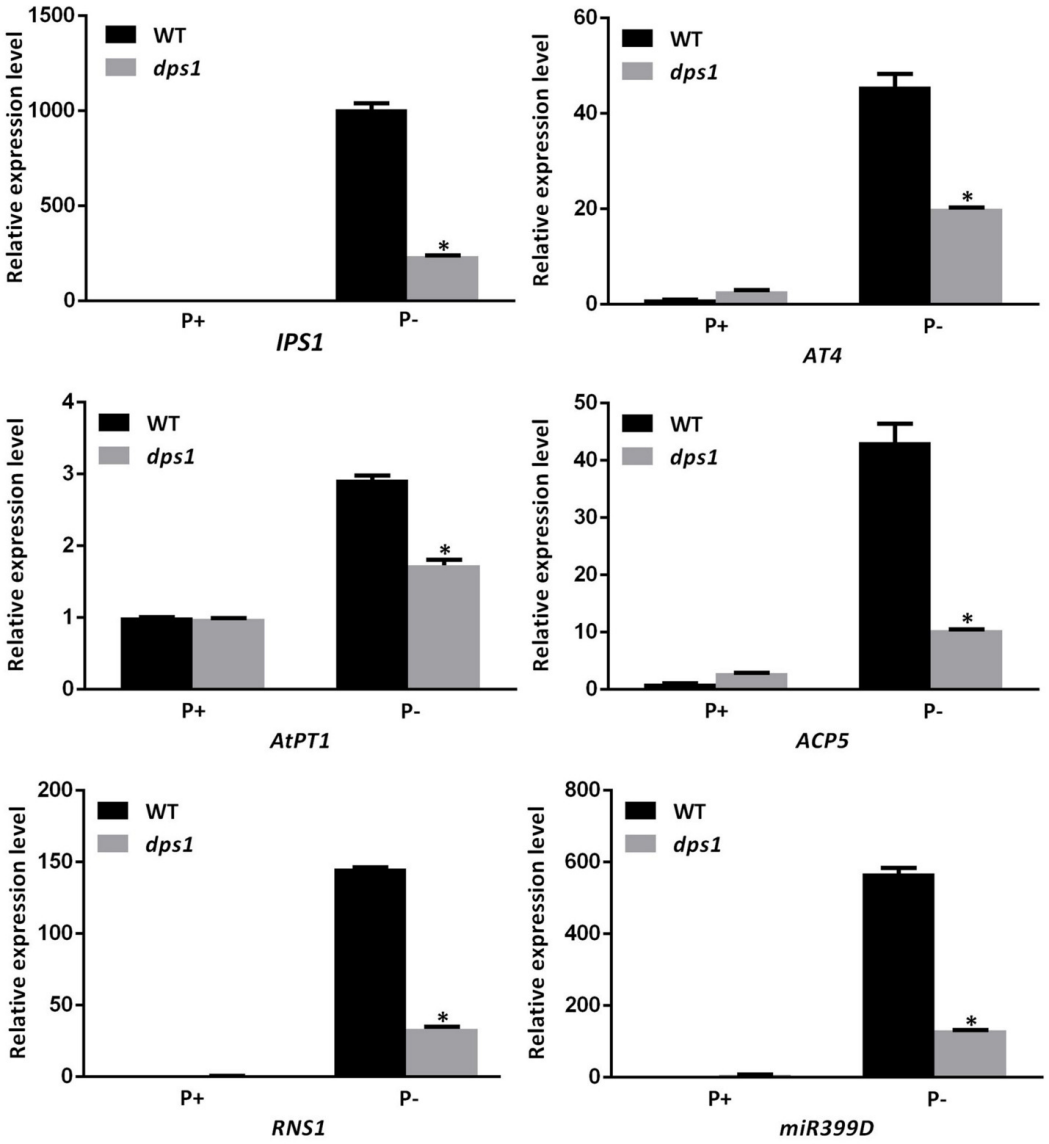
Relative expression of six PSI genes in roots of WT and *dps1* seedlings. Total RNAs extracted from the roots of 7-days-old WT and *dps1* seedlings grown on P+ or P− media were subjected to qPCR analysis. The names of the genes examined are indicated on the bottom of each panel. Three independent experiments were performed with similar results and one representative experiment is shown. Values are means ± SD of three technical replicates and represent fold changes normalized to transcript levels of the WT on P+ medium. An asterisk indicates a significant difference relative to the WT (*t*-test, *P* < 0.05).

To determine whether the *DPS1* mutation affects the expression of other Pi starvation-induced (PSI) genes, we used qPCR to compare the expression levels of six PSI genes in roots and shoots of 7-days-old WT and *dps1* seedlings grown on P+ and P− media. The PSI genes examined included another high-affinity Pi transporter (*AtPT1*), an APase (*ACP5*), two non-coding transcripts *(IPS1* and *At4*), an RNase (*RNS1*), and a microRNA (miR399D) (Sun et al., 2016). The expression levels of these PSI genes did not differ between *dps1* and the WT when plants were grown on P+ medium. The induction of all six PSI genes in both roots and shoots, however, was dramatically lower in *dps1* than in the WT when seedlings were grown under Pi starvation (Figure 2, Supplementary Figure 1). These results suggest that the mutation in *DPS1* might globally affect the expression of PSI genes.

### The *DPS1* mutation impairs the remodeling of RSA induced by Pi starvation

**Figure 3:**
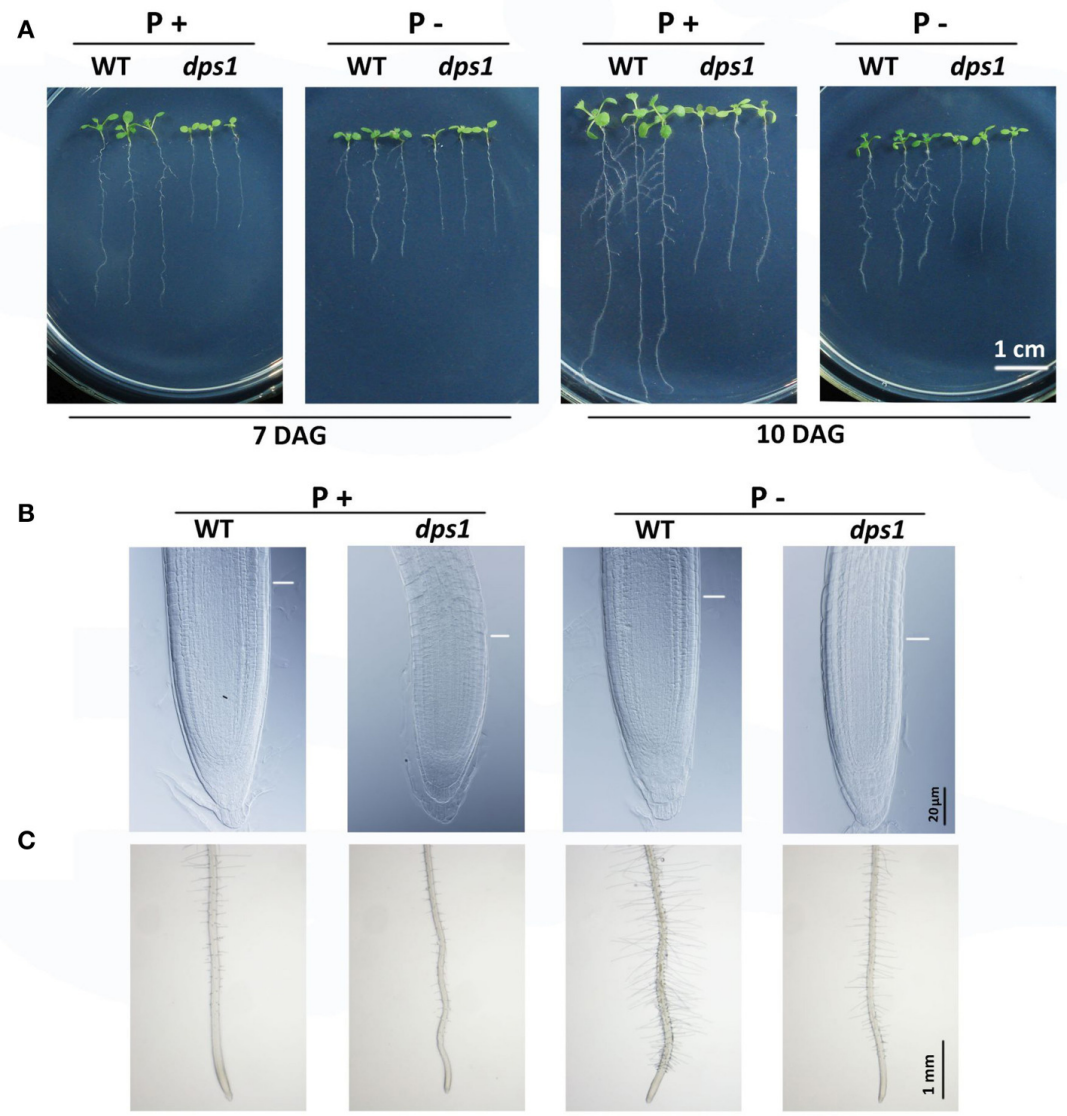
Analyses of RSA for WT and *dps1* seedlings. **(A)** Morphology of 7-days-old and 10-days-old WT and *dps1* seedlings grown on P+ and P− media. **(B)** A close view of root tips of 7-days-old WT and *dps1* seedlings. White lines indicate the boundary between the root meristematic and elongation zones. **(C)** Root hair patterns of 7-days-old WT and *dps1* seedlings grown on P+ and P– media.

The remodeling of RSA is a major developmental response of plants to Pi starvation and includes the inhibition of primary root growth and the promotion of lateral root and root hair formation (Péret et al., 2014). This developmental change increases the root surfaces for Pi uptake. To compare the Pi starvation-induced remodeling of RSA between the WT and *dps1*, we grew seedlings on P+ and P− media and measured the length of the primary root as well as the number of lateral roots on each plant. At 7 and 10 DAG under Pi sufficiency, the length of primary root of *dps1* was about half of that of the WT. When grown under Pi deficiency, the length of primary root of the WT was reduced by approximately 30 and 50% at 7 and 10 DAG, respectively; for *dps1*, however, the length of the primary root was reduced by only about 5 and 20%, respectively (Figure 3A, Supplementary Figure 2A). We then examined the size of the root apical meristem (RAM). As shown in Figure 3B, the RAM was smaller for *dps1* than for the WT under both P+ and P− conditions, suggesting that the cell division activity in the RAM of *dps1* was reduced. Interestingly, the size of the RAM of *dps1* under P− and P+ conditions remained nearly unchanged. At 10 DAG, lateral roots were evident for the WT on P− medium (Figure 3A, Supplementary Figure 2B). Lateral roots, however, were almost completely absent for *dps1* on P− medium. In addition, root hairs were much less abundant and were shorter for *dps1* than for the WT under Pi starvation (Figure 3C). Taken together, these results indicate that the remodeling of RSA induced by Pi starvation is greatly impaired in *dps1*.

### The *DPS1* mutation suppresses the induction of APases and the accumulation of anthocyanins and starch

**Figure 4:**
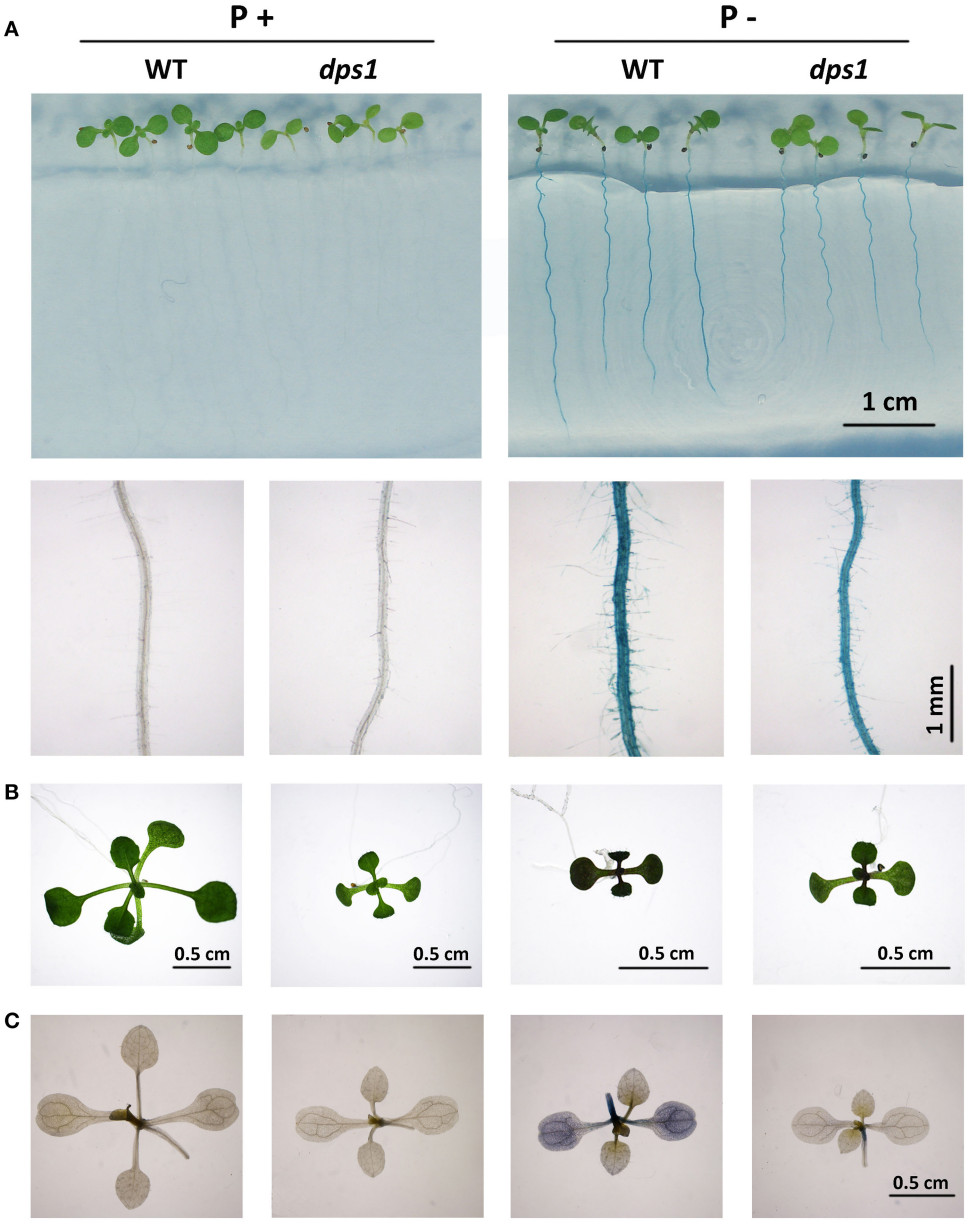
APase activities and accumulation of anthocyanins and starch in WT and *dps1* seedlings. **(A)** Top row, APase activities on the root surface of 7-days-old WT and *dps1* seedlings grown on P+ and P− media as detected by BCIP staining; bottom row, close views of root APase activities of WT and *dps1* seedlings. **(B)** The pictures of 12-days-old WT and *dps1* seedlings grown on P+ and P− media. The accumulation of anthocyanins in P− seedlings are indicated by purple in leaves. **(C)** Starch accumulation in 10-days-old WT and *dps1* seedlings grown on P+ and P− media as indicated by iodine staining.

Induction and secretion of APases in roots and accumulation of anthocyanins and starch in leaves are additional hallmark responses of plants to Pi starvation. The secretion of acid phosphatases and RNases contributes to the increase of environmental Pi concentration by releasing Pi from organophosphates (Tran et al., 2010). The secreted APase activity on the root surface can be detected by applying BCIP (5-bromo-4-chloro-3-indolyl phosphate p-toluidine salt), a substrate for APase, to the root surface (Lloyd et al., 2001). BCIP can be catalyzed by APase to produce a blue precipitate. On P+ medium, no blue staining on the root surface was evident for either the WT or *dps1*. On P− medium, blue staining was evident on the roots of both the WT and *dps1* but the intensity of blue staining was reduced on *dps1* (Figure 4A).

Accumulation of anthocyanins in leaves is believed to be photoprotective for the chloroplast membranes under Pi deficiency (Hernández and Munné-Bosch, 2015). We therefore compared the accumulation of anthocyanins in leaves of Pi-deficient WT and *dps1* plants. Anthocyanin accumulation was higher in the cotyledons and true leaves of 12-days-old WT seedlings grown on P− medium than on P+ medium (Figure 4B). For *dps1* seedlings grown on P− medium, however, anthocyanin accumulation was not evident, i.e., neither the cotyledons nor true leaves became purple. Quantitative analysis of anthocyanin contents confirmed this result (Supplementary Figure 3). We also used qPCR to measure the expression level of eight genes involved in anthocyanin biosynthesis and regulation. The anthocyanin biosynthetic genes examined encode a phenylalanine ammonia-lyase (PAL); an anthocyanidin synthase (ANS); a dihydroflavonol reductase (DFR); and a chalcone synthase (CHS) (Falcone Ferreyra et al., 2012). The regulatory genes for anthocyanin biosynthesis included *PAP1, GL3, EGL3*, and *TTG1* (Zhang et al., 2003). The results showed that the expression levels of all genes examined except *TTG1* were upregulated in the WT by Pi starvation. Such upregulation, however, was significantly reduced in *dps1* (Supplementary Figure 4). These results suggest that the decreased accumulation of anthocyanins in *dps1* was partly due to the reduced transcription of the genes involved in anthocyanin biosynthetic and regulatory pathways.

The accumulation of starch in Arabidopsis leaves was assessed by iodine staining. There was a dark black staining in the leaves of the WT grown on P− medium but a much lighter staining in *dps1*, indicating that Pi starvation-induced starch accumulation was strongly repressed in *dps1* (Figure 4C).

Maintenance of Pi homeostasis is critical for plant growth and development. The Pi contents of shoots and roots were lower in the *dps1* mutant than in the WT under P+ conditions. In contrast, the Pi contents of shoots and roots were higher in *dps1* than in the WT under P− conditions (Supplementary Figure 5A). The trends were similar for levels of total P content (Supplementary Figure 5B). These results indicate that Pi homeostasis is perturbed in *dps1*.

In a summary, a mutation in the *DPS1* gene globally reduced plant sensitivity to Pi starvation.

### *DPS1* encodes a chloroplast-localized pseudouridine synthase

**Figure 5:**
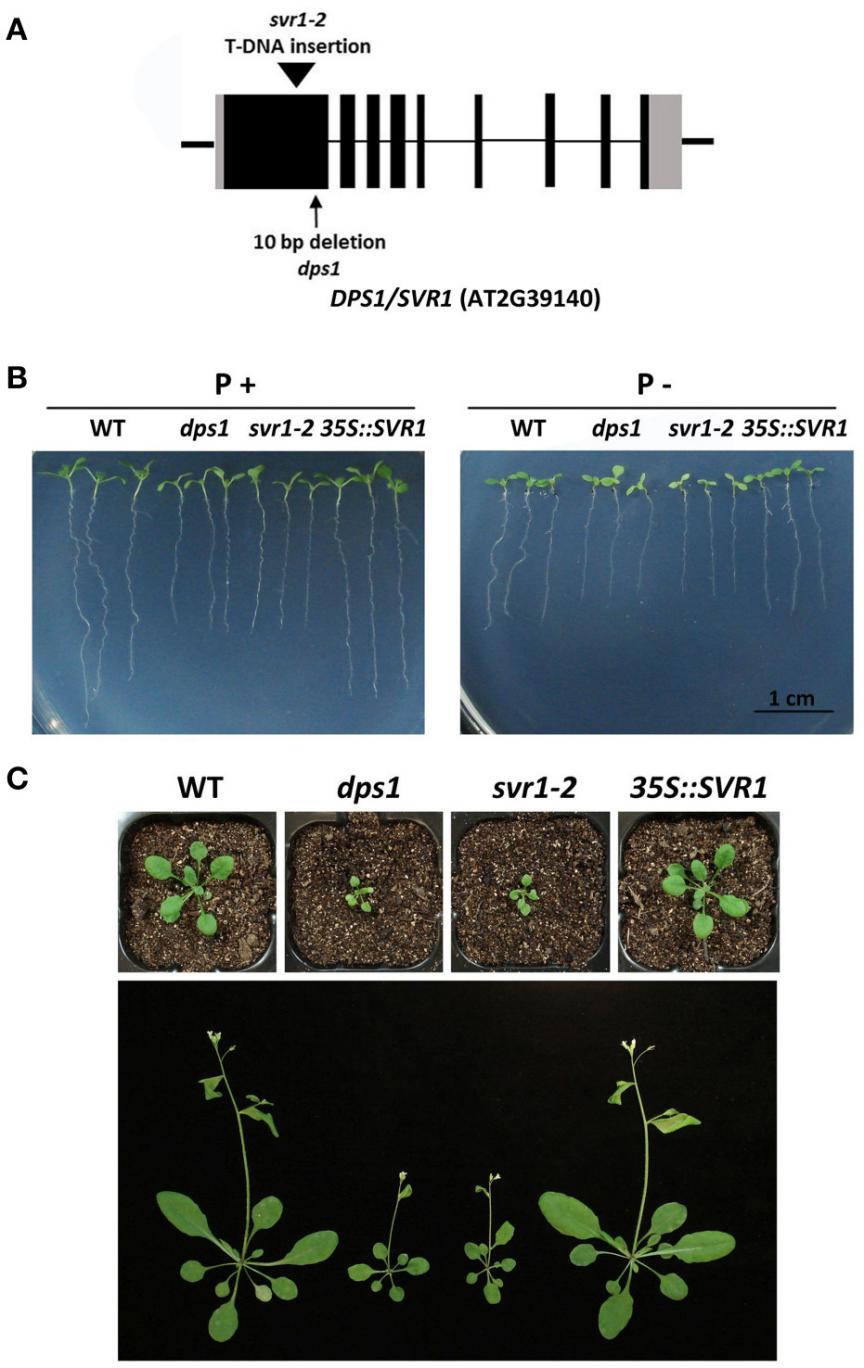
Molecular identification and genetic complementation of the *DPS1* gene. **(A)** Diagram showing the position of the T-DNA insertion in *svr1-2* and the 10-bp deletion in *dps1*. Black box: exons; gray box: 5′ and 3′ UTRs; thin line: introns; thick line: flanking sequences surrounding the *DPS1* gene. **(B)** Morphology of 7-days-old seedlings of the WT, *dps1, svr1-2*, and one complementation line grown on P+ and P− media. **(C)** Morphology of the WT, *dps1, svr1-2*, and one complementation line grown in soil. Top, 21-days-old plants; bottom, 35-days-old plants.

To identify the molecular lesion in *dps1*, we backcrossed *dps1* to the WT. All F_1_ progeny showed WT phenotypes. In the F_2_ progeny derived from selfed F_1_ plants, the *dps1* mutant phenotypes segregated with a 1:3 ratio, indicating that the *dps1* phenotypes were caused by a single recessive mutation. A set of molecular markers was used to roughly map the position of the mutation to a 2.5 MB region on chromosome 2. Combined with the whole-genome sequencing technique, we found a deletion of 10 bp in the first exon of the AT2G39140 gene, which encodes a chloroplast-localized pseudouridine (Ψ) synthase, SVR1 (Yu et al., 2008). This deletion caused a frameshift that generated a stop codon, resulting in a premature termination of protein translation (Figure 5A). A WT *SVR1* gene under the control of the *CAMV 35S* promoter was then introduced to the *dps1* mutant. We grew the WT, *dps1, svr1-2* (Yu et al., 2008), and the complementation line on P+ and P− media. At 7 DAG, the morphology of *svr1-2* was similar to that of *dps1*, while the morphology of the complementation line was similar to that of the WT (Figure 5B). In addition, *svr1-2* grown on P− medium also exhibited light blue BCIP staining on its root surface, and the reduced BCIP staining in *dps1* was restored to the level of the WT in the complementation line (Supplementary Figure 6). The WT, *dps1, svr1-2*, and the complementation line were then grown in soil to maturity. After growing for 3 weeks (Figure 5C, upper panel) and 5 weeks (Figure 5C, bottom panel), *dps1* plants were small and had pale green leaves, suggesting a reduced photosynthetic activity. These growth defects were also evident for *svr1-2*. In contrast, the morphology of the complementation line was similar that of the WT (Figure 5C). These results demonstrate that the mutant phenotypes of *dps1* were caused by the mutation in the *SVR1* gene.

A previous study reported that a mutation of SVR1 causes defects in chloroplast rRNA processing; as a result, the accumulation of several chloroplast-encoded proteins, including the large subunit of Rubisco (RbcL), was reduced in *svr1-2* (Yu et al., 2008). We also confirmed that the accumulation of RbcL in *dps1* is greatly reduced (Supplementary Figure 7). This provided another line of evidence that the mutation of the *SVR1* gene was responsible for the mutant phenotypes of *dps1*.

### Impaired chloroplast translation decreases plant sensitivity to Pi starvation

**Figure 6:**
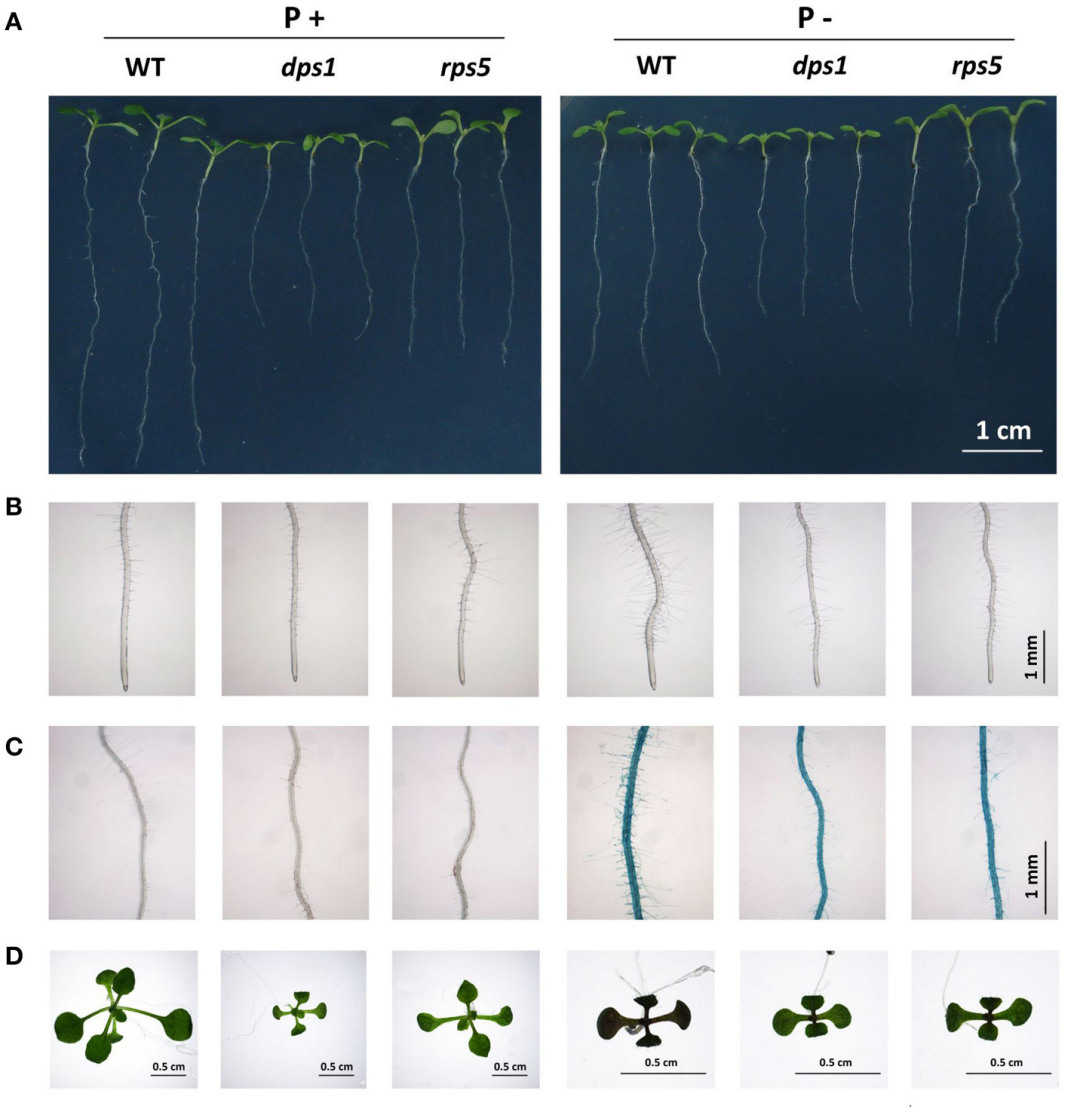
Primary root growth, root hair formation, APase activities, and anthocyanin accumulation in the WT and *rps5* grown on P+ and P− media. **(A)** Morphology of WT and *rps5* seedlings. **(B)** Root hair patterns of WT and *rps5* seedlings. **(C)** APase activities on the root surface of WT and *rps5* seedlings as detected by BCIP staining. **(D)** Anthocyanin accumulation in WT and *rps5* seedlings as indicated by purple in leaves. In **(A–C)**, the seedlings were 7 days old; in **(D)**, the seedlings were 12 days old.

The analyses of *dps1* and the previously reported *svr1-2* mutant suggested that impaired chloroplast protein translation might decrease plant sensitivity to Pi starvation. We therefore examined the Pi responses of another Arabidopsis mutant, *rps5*. This mutant contains a mutation in the plastid ribosomal protein S5 (RPS5), which dramatically reduces the abundance of chloroplast 16S rRNA and severely impairs 16S rRNA processing; as a result, this mutation reduces chloroplast protein translation (Zhang et al., 2016). The morphologies of 7-days-old *rps5* grown on P+ and P− media were similar to those of *dps1* (Figure 6A). Like *dps1*, the length of the primary root of *rps5* under Pi sufficiency was 60% of that of the WT. Under Pi deficiency, the primary root length was reduced by 30% for the WT but by only 5% for both *rps5* and *dps1*. Similarly, root hair formation (Figure 6B), root surface-associated APase activity (Figure 6C), and anthocyanin accumulation (Figure 6D) were lower in *rps5* than in the WT under Pi deficiency. We then used qPCR to analyze the expression levels of four PSI genes including *IPS1, At4, AtPT1*, and *ACP5* in 7-days-old WT and *rps5* seedlings grown on P+ and P− media. On P+ medium, the expression levels of these four PSI genes did not significantly differ between *rps5* and the WT. On P− medium, however, expression levels of these PSI genes were much lower in *rps5* than in the WT (Supplementary Figure 8). These results indicate that the *rps5* mutant also has decreased sensitivity to Pi starvation.

**Figure 7:**
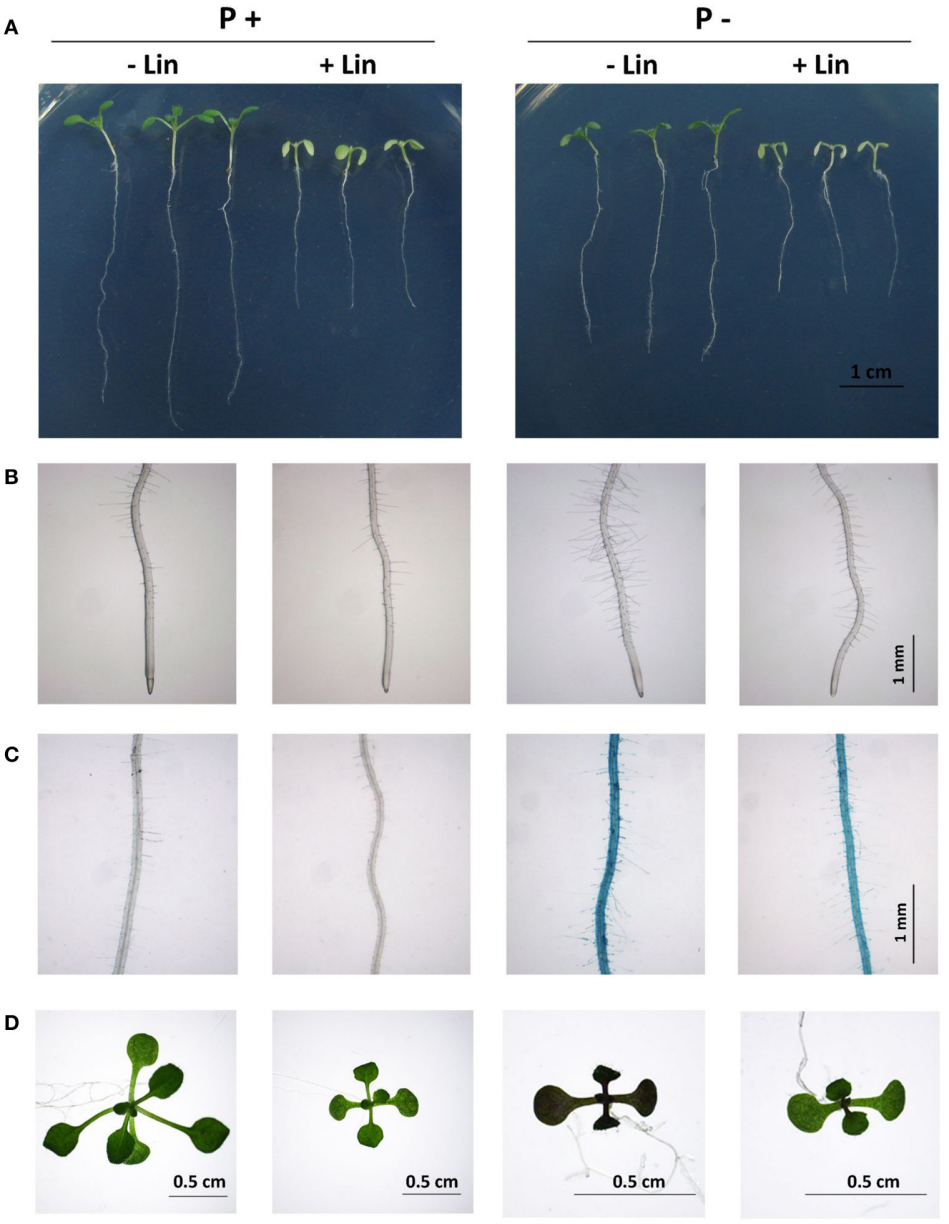
Primary root growth, root hair formation, APase activities, and anthocyanin accumulation in WT seedlings without (−Lin) or with (+Lin) lincomycin treatment. **(A)** Morphology of −Lin and +Lin seedlings. **(B)** Root hair patterns of −Lin and +Lin seedlings. **(C)** APase activities on the root surface of −Lin and +Lin seedlings as detected by BCIP staining. **(D)** Anthocyanin accumulation in −Lin and +Lin seedlings. In **(A–C)**, the seedlings were 7 days old, and the concentration of lincomycin was 30 μM; in **(D)**, the seedlings were 12 days old, and the concentration of lincomycin was 15 μM.

To confirm that the defects in chloroplast protein translation reduce plant sensitivity to Pi starvation, we treated WT plants with lincomycin, a chemical that specifically inhibits chloroplast protein translation (Sullivan and Gray, 1999). Arabidopsis seeds were directly sown on P+ and P− media with or without 30 μM lincomycin. At 7 DAG, the WT seedlings grown on lincomycin-containing P+ medium had short primary roots and pale-green leaves, i.e., phenotypes that mimic those of *dps1* and *rps5* (Figure 7A). On P− medium, the inhibition of primary root growth and of root hair formation of the lincomycin-treated seedlings was largely blocked, which was similar to the responses of *dps1* and *rps5* to Pi starvation (Figures 7A,B). Furthermore, the induction of root surface-associated APase activity, the accumulation of anthocyanins in leaves, and the expression of PSI genes induced by Pi starvation were greatly reduced in the lincomycin-treated WT seedlings (Figures 7C,D and Supplementary Figure 9).

## Discussion

To cope with Pi starvation, plants display an array of adaptive responses. These responses enhance the plant's ability to acquire, transport, and remobilize Pi. How plant cells perceive a change in Pi availability in the environment and then trigger these adaptive responses has been extensively studied, and several key molecular components involved in regulating plant responses to Pi starvation have been identified. However, whether the status of a specific organelle can affect the magnitude of plant responses to Pi starvation remains unknown.

In this study, we identified an Arabidopsis mutant, *dps1*, with greatly reduced sensitivity to Pi starvation (Figures 1, 2). This mutant is almost completely insensitive to Pi starvation in the remodeling of RSA (Figure 3). The induction of APase activity on the root surface and the accumulation of anthocyanins and starch in leaves are partially impaired (Figure 4). The transcriptional responses in both shoots and roots are also reduced (Figure 2 and Supplementary Figure 1). Consequently, Pi homeostasis is perturbed in *dps1* (Supplementary Figure 5). Our genetic and molecular analyses indicate that the decreased sensitivity to Pi starvation in *dps1* is caused by a mutation in a chloroplast-localized Ψ synthase, SVR1 (Figure 5).

Ψ is the most common minor base in rRNAs and tRNAs. In higher plants, chloroplast rRNAs are transcribed from the chloroplast genome, and the Ψs within the chloroplast rRNAs are produced from uridines through isomerization catalyzed by a Ψ synthase. In the *svr1-2* mutant, chloroplast rRNA processing is dramatically impaired, i.e., the maturation of 23S, 16S, 5S, and 4.5S rRNAs is largely suppressed (Yu et al., 2008). This defect might affect the assembly of chloroplast ribosomes, which would reduce chloroplast translation. Indeed, the accumulation of three chloroplast genome-encoded proteins, D1, ATPα, and RbcL, are greatly reduced in *svr1-2*. These three proteins are components of the photosynthesis machinery. A pulse-labeling experiment further confirmed that the reduced accumulation of these proteins was due to the reduced efficiency of protein translation (Yu et al., 2008). A reduction in chloroplast translation could, in turn, reduce photosynthesis in *svr1-2* and thereby explain the mutant's pale-green leaves and retarded shoot growth. These mutant growth phenotypes were also observed in *dps1*.

Many previous studies have indicated that a mutation of a specific chloroplast ribosomal protein could affect plant growth, development, and metabolism, including embryogenesis, leaf patterning, female gametophyte development, seed germination, seedling development, and photosynthesis (Pesaresi et al., 2001; Morita-Yamamuro et al., 2004; Bryant et al., 2011; Szakonyi and Byrne, 2011; Romani et al., 2012; Gong et al., 2013; Zsögön et al., 2014). In most of these cases, the research demonstrated that chloroplast translation was impaired in the mutants. To date, however, only a few reports showed that impaired chloroplast translation affects plant responses to abiotic stress. These include that knockout of the Arabidopsis chloroplast ribosomal protein *RPS5* gene and the tobacco chloroplast ribosomal protein *RPL33* gene reduced plant tolerance to cold stress (Rogalski et al., 2008; Zhang et al., 2016), and downregulation of the Arabidopsis chloroplast ribosomal protein *RPS1* gene increased plant sensitivity to heat stress (Yu et al., 2012). Our study of *dps1*/*svr1* mutants provides the first example of an impairment in chloroplast translation desensitizing plants to Pi starvation. To provide more evidence for this notion, we compared all hallmark responses to Pi starvation between *rps5* and *dps1*. *rps5* contains a mutation in the chloroplast ribosomal protein S5 (Zhang et al., 2016). Using iTRAQ-based method, the researchers globally compared the change of proteins levels between the WT and *rps5*. They found that 294 proteins were differentially expressed in *rps5*, including nine down-regulated chloroplast genome-encoded proteins. Indeed, like *dps1, rps5* also showed reduced responses to Pi starvation. Furthermore, using a chemical inhibitor of chloroplast protein translation, we demonstrated that when chloroplast protein translation is blocked, plants become almost completely insensitive to Pi starvation.

The mechanism by which impaired chloroplast translation desensitizes plants to Pi starvation is unknown. We reason that impaired chloroplast translation interferes with the assembly of photosynthetic apparatuses PSI and PSII, as well with as the production of enzymes involved in the Calvin cycle. These defects may greatly reduce photosynthetic activity, which requires the input of large amounts of Pi to generate ATP and other phosphorylated sugar intermediates. When photosynthesis is suppressed, the demand of plants for Pi is decreased. This in turn, causes plants to slow the processes involved in Pi acquisition, transport, and remobilization, therefore reducing their responses to Pi starvation. In contrast, elevation of CO_2_ levels in atmosphere is well known to enhance photosynthesis in leaves and increase the allocation of carbon to roots. The increase of carbon in roots stimulates root growth. Both increased photosynthesis in leaves and enhanced root growth increase the plant's demand for Pi input from the environment (for review, see Jin et al., 2015). To acquire more Pi from the rhizosphere, plants grown under elevated CO_2_ conditions activate root exudation of organic acids and APases, a process that mimics the response to Pi starvation. And, root exudation is further increased when plants were exposed to both Pi deficiency and elevated CO_2_ conditions, which had been observed in multiple plant species (Campbell and Sage, 2006; Haase et al., 2007; Niu et al., 2013; Pandey et al., 2015). This phenomenon can also be interpreted as that the increased photosynthesis in leaves enhances plant responses to Pi starvation. Combined these published results with our study presented here, we propose that chloroplast translation, which may affect plant demand for input of external Pi, is an important determinant for plant sensitivity to Pi starvation. Also, as a consequence of altered demand for input of external Pi, Pi homeostasis could not be maintained in *dps1* as the WT under both P+ and P− conditions (Supplementary Figure 5). This might explain why growth and development of *dps1* on P+ medium was also affected.

Our hypothesis is also consistent with that reported by Lai et al. (2007). To investigate the factors that determine plant Pi sensitivity, Lai et al used the level of the induction of PSI genes as an indicator of the level of plant response to Pi starvation. They found that cell division activity, but not cell expansion rate, is correlated with the magnitude of plant responses to Pi starvation. To investigate the role of cell division, Lai et al. treated plants with cytokinin, which increases cell division and shoot growth but inhibits root growth. The cytokinin-treated plants displayed high Pi-starvation responses in shoots but low Pi-starvation responses in roots. Lai et al. also found that salt treatment, which reduces cell division in the RAM, reduces Pi-starvation responses. The authors proposed that actively growing tissue has a high demand for Pi and therefore has an enhanced response to Pi starvation.

How does the level of Pi demand in chloroplast determine the magnitude of plant responses to Pi starvation? Previous studies have indicated that the chloroplast and nucleus communicate via retrograde signaling (Chan et al., 2016). Therefore, a change in chloroplast translation might be communicated to the nucleus through retrograde signals, which could change the expression of nuclear genes and thereby adjust cellular activities. Several molecules that may be involved in the retrograde signaling have been identified, such as reactive oxygen species and the plastid metabolites, methylerythritol cyclo-diphosphate and tetrapyrrole (Chan et al., 2016). When protein translation is impaired in chloroplasts, which subsequently reduces photosynthesis and Pi demand in leaves, chloroplasts might send a signal to the nucleus to suppress the transcription of nuclear genes involved in photosynthesis and in Pi signaling, transport, and utilization. The decreased photosynthesis in leaves may also generate a signal that is translocated to roots to reduce root Pi responses by affecting gene expression in roots. These reduced root responses include the remodeling of RSA and the induction of root-associated APase activity (Figures 3, 4). In support of this possibility, our analyses of PSI marker genes, which are involved in Pi signaling, transport, and remobilization, indicated that the transcriptional responses in P− *dps1* roots are greatly suppressed (Figure 2). In other words, the change of Pi demand in chloroplasts modulates plant sensitivity to Pi starvation through actively reprogramming of gene expression in both shoots and roots.

In summary, we identified an Arabidopsis mutant, *dps1*, with greatly reduced sensitivity to Pi starvation. Our genetic, molecular, and pharmacological analyses suggest that the reduced Pi sensitivity of *dps1* results from impaired chloroplast protein translation, which subsequently reduces photosynthetic activity. Together with previous reports that the increased photosynthetic activity induced by elevated atmospheric CO_2_ enhances root responses to Pi starvation, we propose that chloroplast translation is an important determinant of plant sensitivity to Pi starvation. And, the magnitude of plant responses to Pi starvation might depend on the balance between the level of Pi demand of plants and availability of Pi in the environment.

## Author contributions

SL and DL conceived and designed the experiments. SL, CL, and ZZ carried out the experiments. SL, YZ, and DL analyzed the data. SL and DL wrote the manuscript.

### Conflict of interest statement

The authors declare that the research was conducted in the absence of any commercial or financial relationships that could be construed as a potential conflict of interest.

## Abbreviations

APase: acid phosphatase
BCIP: 5-bromo-4-chloro-3-indolyl-phosphate
iTRAQ: isobaric tags for relative and absolute quantification
LUC: luciferase
P: phosphorus
Pi: phosphate
PSI: phosphate starvation-induced
qPCR: quantitative real-time PCR
RAM: root apical meristem
RSA: root system architecture
Ψ: pseudouridine.
