# Supplementary material for: Functional Disruption of a Chloroplast Pseudouridine Synthase Desensitizes Arabidopsis Plants to Phosphate Starvation
Source: Front Plant Sci. 2017 Aug 15;8:1421. doi: 10.3389/fpls.2017.01421 (PMC5559850; doi:10.3389/fpls.2017.01421)
Supplement: Supplementary file 1 [file Table1.DOCX]

**Supplementary Table 1.** The sequences of the primers used for qPCR analyses

| **Primer name** | **Primer sequence（5’ to 3’）** | |
| --- | --- | --- |
| AT3G18780-Actin2-realtime-F | | GACCTTGCTGGACGTGACCTTAC |
| AT3G18780-Actin2-realtime-R | | GTAGTCAACAGCAACAAAGGAGAGC |
| AT5G03545-At4-realtime-F | | TGGCCCCAAACACAAGAG |
| AT5G03545-At4-realtime-R | | CGAACATTCACAATCATAATCTCC |
| AT3G09922-IPS1-realtime-F | | AGACTGCAGAAGGCTGATTCAGA |
| AT3G09922-IPS1-realtime-R | | TTGCCCAATTTCTAGAGGGAGA |
| AT5G43350-AtPT1-realtime-F | | TGATGATCTTGTGCTCTGTCG |
| AT5G43350-AtPT1-realtime-R | | ATGACACCCTTGGCTTCGT |
| AT2G38940-AtPT2-realtime-F | | CGAAGCTCCTCGGTCGTAT |
| AT2G38940-AtPT2-realtime-R | | GGAGAGTCCCAGGCTTTTGT |
| AT2G02990-RNS1-realtime-F | | TTGTTATCCAAATTCAGGCAAA |
| AT2G02990-RNS1-realtime-R | | AGTTAGGCCAAAGACCATGAAT |
| AT2G34202-miR399D-realtime-F | | AATACTCCTATGGCAGATCGCATTGG |
| AT2G34202-miR399D-realtime-R | | TCCTTTGGCAGAGAAGCATTTTACTTG` |
| AT3G17790-ACP5-realtime-F | | CTTAAGTCCTATTGCAGGCTAGGT |
| AT3G17790-ACP5-realtime-R | | TTGCTAAAAATGATAGGGATGCT |
| AT2G37040-PAL1-realtime-F | | CTTGGAACAGAGCTTTTGACCG |
| AT2G37040-PAL1-realtime-R | | CGTGAAAACCTTGTCGAACTCTTC |
| AT4G22880-ANS-realtime-F | | TCACACCGATGTAAGCGCTTT |
| AT4G22880-ANS-realtime-R | | CCCATTTGCCCTCGTAGAAA |
| AT5G13930-CHS-realtime-F | | CGCATCACCAACAGTGAACAC |
| AT5G13930-CHS-realtime-R | | CGTTTCCGAATTGTCGACTTG |
| AT5G42800-DFR-realtime-F | | TTGGTGGTCGGTCCATTCAT |
| AT5G42800-DFR-realtime-R | | GCCTCGTTCCGAGTGATAGG |
| AT1G56650-PAP1-realtime-F | | TCGACCTCGATCCTTCACAGT |
| AT1G56650-PAP1-realtime-R | | GGCATGGAGGATTAACGTCAAC |
| AT5G24520-TTG1-realtime-F | | TTCCTCCGTCTTTGGGAAATT |
| AT5G24520-TTG1-realtime-R | | TCGCTCGTTTTGCTGTTGTT |
| AT1G63650-EGL3-realtime-F | | GAAACCGCCGATAGCAAAGTC |
| AT1G63650-EGL3-realtime-R | | AACGGGAAGCAAACCACTGT |
| AT5G41315-GL3-realtime-F | | AGATTCTAGGCGACGAGATTTACG |
| AT5G41315-GL3-realtime-R | | GTTGGTAGTTCTGCTCGGAGAAG |
