# Supplementary material for: Functional Disruption of a Chloroplast Pseudouridine Synthase Desensitizes Arabidopsis Plants to Phosphate Starvation
Source: Front Plant Sci. 2017 Aug 15;8:1421. doi: 10.3389/fpls.2017.01421 (PMC5559850; doi:10.3389/fpls.2017.01421)
Supplement: Supplementary file 2 [file Table2.DOCX]

**Supplementary Table 2.** The sequences of the markers used for molecular mapping.

| **Primer name** | **Primer sequence（5’ to 3’）** | |
| --- | --- | --- |
| 1T-F | | TGAAGTGGGATGAATTCTTCC |
| 1T-R | | CTATGGGTCTGACTGGTAGTC |
| 1M-F | | GTCAAGATGGGCTGCCATATTATC |
| 1M-R | | CAGGAAGTCCACCGTCAGAAG |
| 1B-F | | ACACCAACCCTAAACCTCTCC |
| 1B-R | | CCTATGAATTCGCCCAATGGC |
| 2T-F | | CCTCGATCTTTATCGCTATGATCC |
| 2T-R | | AATACTTCATAAGGAGGTAGGTAC |
| 2M-F | | ATGCACGCACCCTTCTACTC |
| 2M-R | | TTCGCACATGTGAGATTATGGG |
| 2B-F | | ACGAATATTGATTGTCTAAG |
| 2B-R | | AACCTAAGGGAAGGCTAC |
| 3T-F | | TAGTAGATCCGCCCCTGGAC |
| 3T-R | | GGTTGGTTTGTCGTTACTATGTG |
| 3M-F | | CCCCGAGTTGAGGTATT |
| 3M-R | | GAAGAAATTCCTAAAGCATTC |
| 3B-F | | CCAGCACATCTTGAAGTTCC |
| 3B-R | | GCATTCCTCTCATCATAGATCC |
| 4M-F | | CTCGAGAGCCACTTTAGCTTTC |
| 4M-R | | AGTGCTGATTGGAGAGGTGC |
| 4B-F | | ATCCGGCTGGAACTTGAGAC |
| 4B-R | | TTACTCTCCCGGTGGTCTTC |
| 5T-F | | CGGTAATACCTATGGAGCCGCCG |
| 5T-R | | GCGCATGGTACCGCTAATGGCAG |
| 5M-F | | AAACTCGAGAGTTTTGTCTAGATC |
| 5M-R | | CTCAGAGAATTCCCAGAAAAATCT |
| 5B-F | | ATGTTGTGTATCAGTCCACG |
| 5B-R | | GATTTAAAGCCTGATTGGCTG |
| T26B15-F | | TGGATTTTGAACAAAGTTAC |
| T26B15-R | | CAATCTCAGCCTCATTGG |
| T16B24-F | | ATGAACGGAGTAGCTATC |
| T16B24-R | | CGCGTAGAACATAATCTGTA |
