# Supplementary material for: Functional Disruption of a Chloroplast Pseudouridine Synthase Desensitizes Arabidopsis Plants to Phosphate Starvation
Source: Front Plant Sci. 2017 Aug 15;8:1421. doi: 10.3389/fpls.2017.01421 (PMC5559850; doi:10.3389/fpls.2017.01421)
Supplement: Supplementary file 3 [file DataSheet1.docx]

*Supplementary figures*

Functional disruption of a chloroplast pseudouridine synthase desensitizes Arabidopsis plants to phosphate starvation

Shan Lu, Chenyi Li, Ye Zhang, Zai Zheng, and Dong Liu^🞰^

🞰 Correspondence:

Dong Liu, School of Life Sciences, Tsinghua University, Beijing 100084, China

Email: [liu-d@tsinghua.edu.cn](mailto:liu-d@tsinghua.edu.cn)


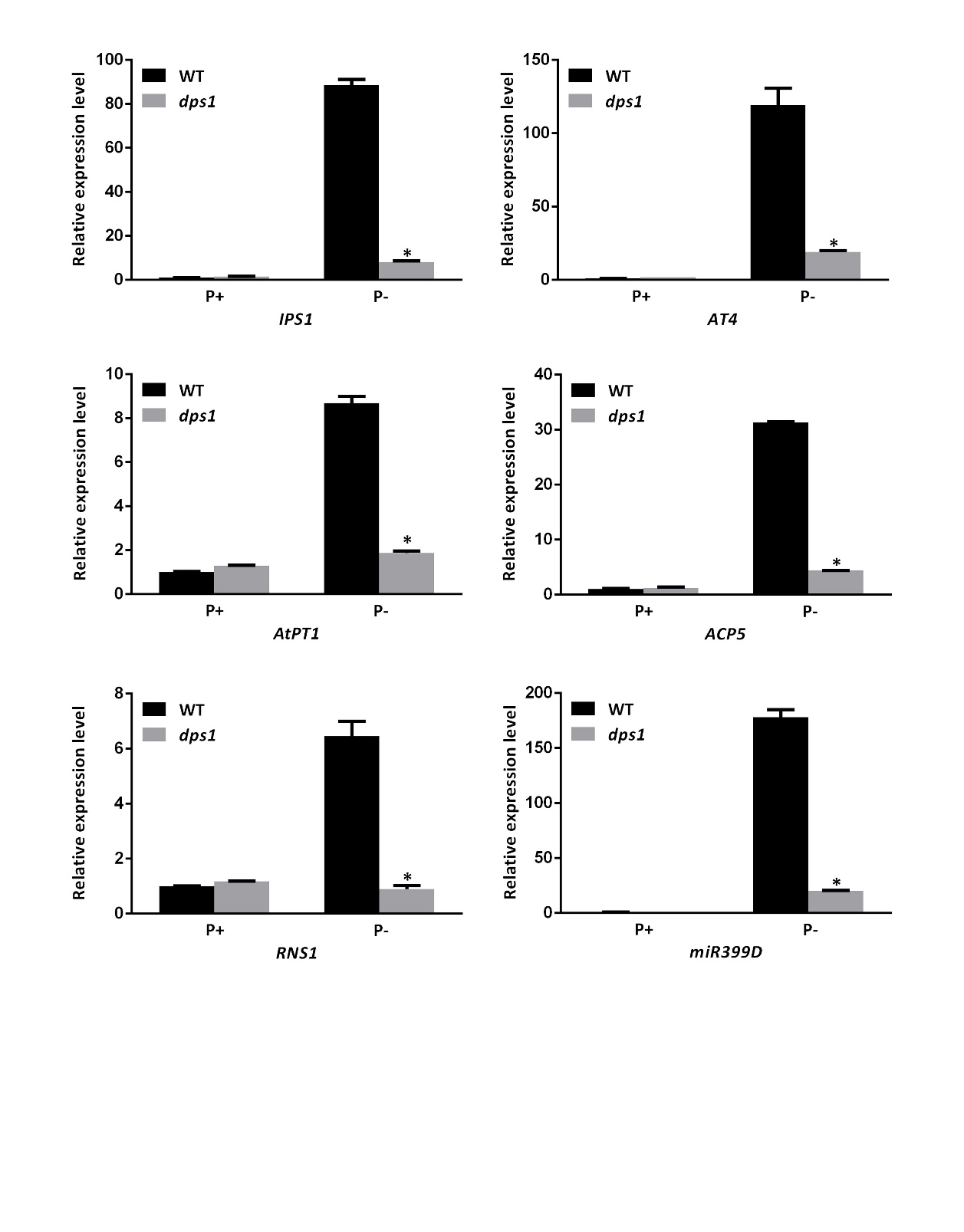
**Supplementary Figure 1**

Supplementary Figure 1. Relative expression of six PSI genes in shoots of WT and *dps1* seedlings. Total RNAs extracted from the shoots of 7-d-old WT and *dps1* seedlings grown on P+ or P- media were subjected to qPCR analysis. The names of the genes examined are indicated on the bottom of each panel. Three independent experiments were performed with similar results and one representative experiment is shown. Values are means ± SD of three technical replicates and represent fold changes normalized to transcript levels of the WT on P+ medium. An asterisk indicates a significant difference relative to the WT (*t*-test, P < 0.05).

**Supplementary Figure 2**


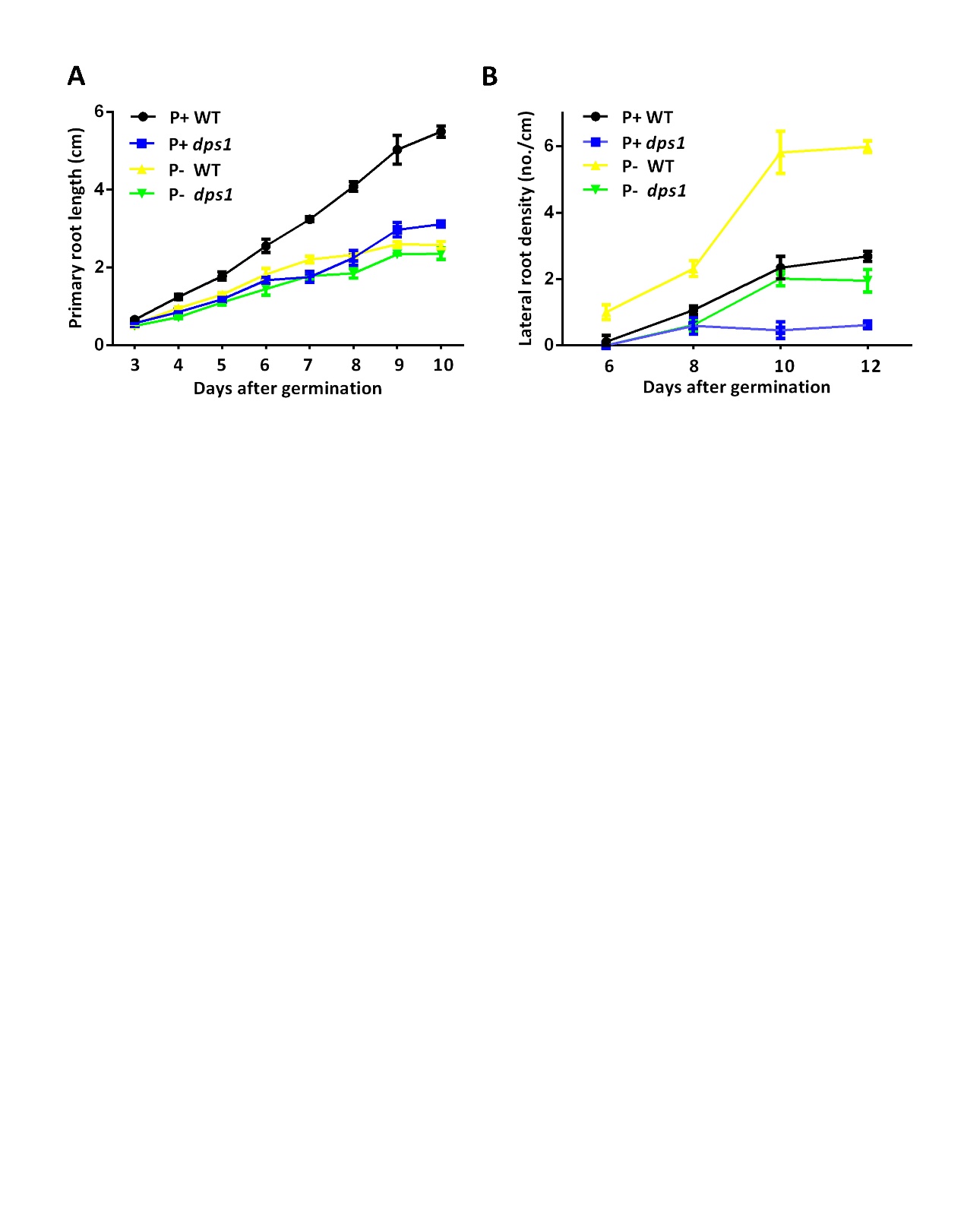


Supplementary Figure 2. Root growth of WT and *dps1* seedlings grown on P+ and P- media. (A) Changes in primary root length from 3 to 10 DAG. (B) Changes in lateral root density from 6 to 12 DAG. In (A, B), Three independent experiments were performed with similar results and one representative experiment is shown. Values are means ± SD of three technical replicates (n ≥ 20).


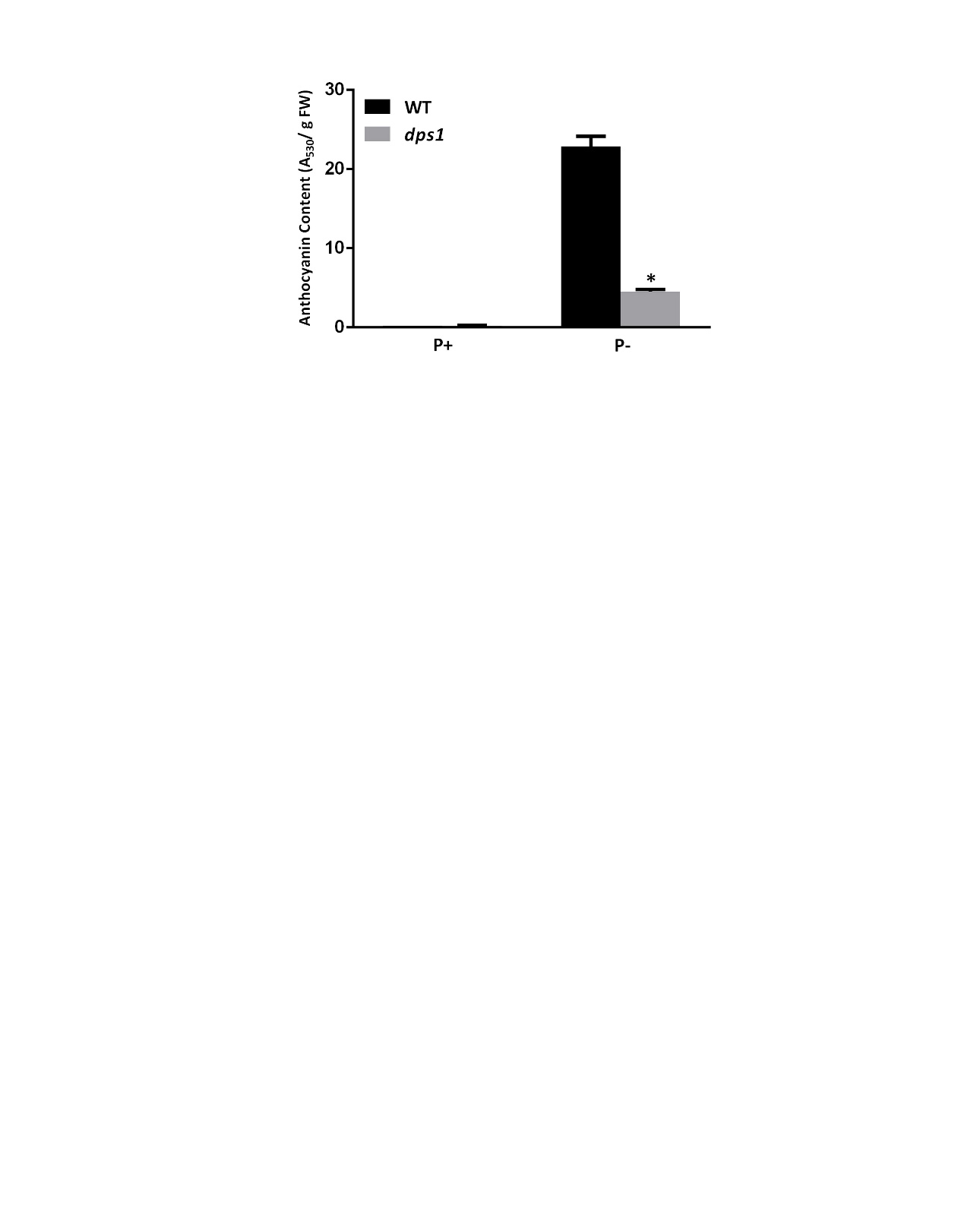
**Supplementary Figure 3**

Supplementary Figure 3. Anthocyanin contents in 12-d-old WT and *dps1* seedlings grown on P+ and P- media. Three independent experiments were performed with similar results and one representative experiment is shown. Values are means ± SD of three technical replicates (*t*-test, P < 0.05). An asterisk indicates a significant difference between *dps1* and the WT.

**Supplementary Figure 4**


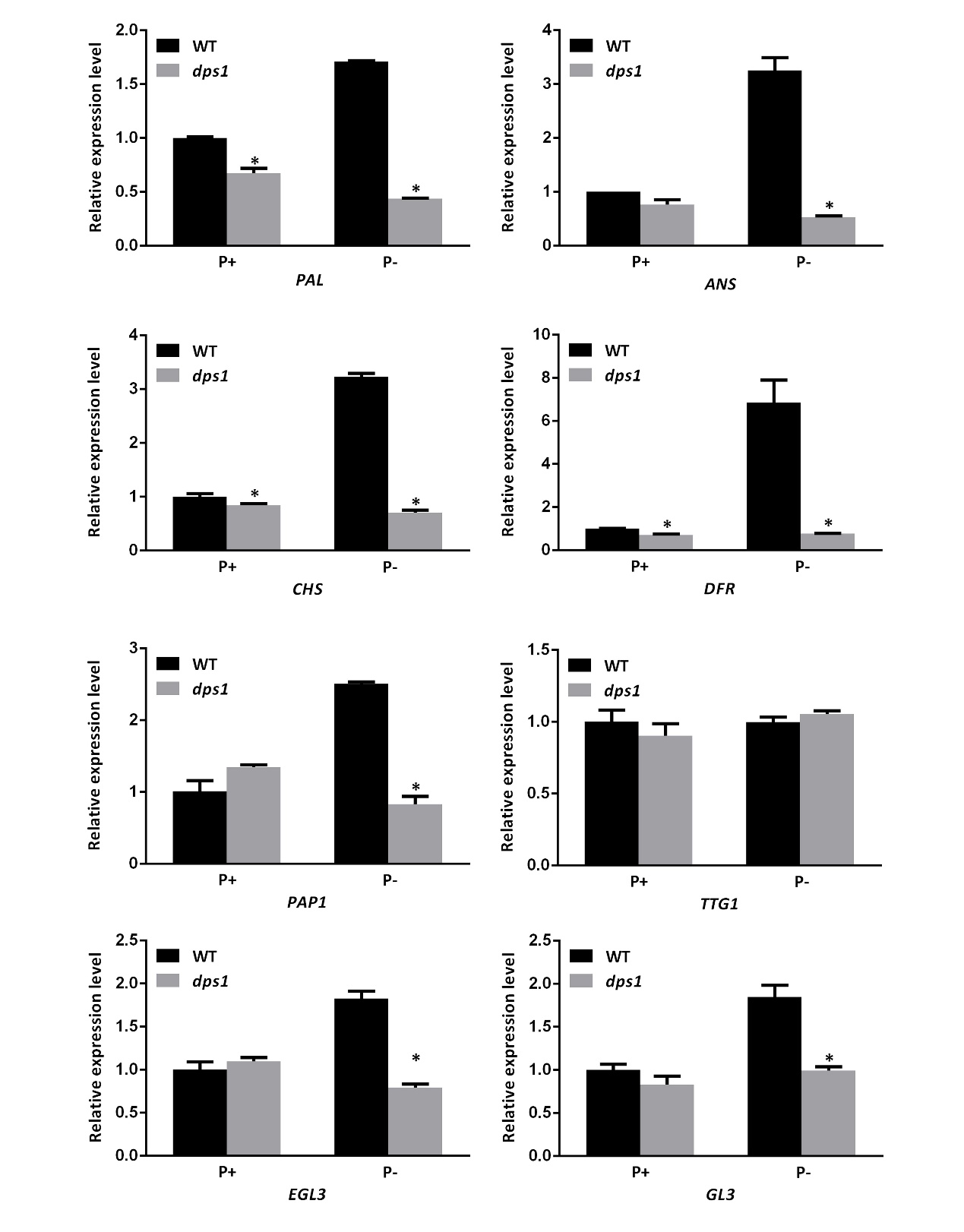


Supplementary Figure 4. Relative expression of eight anthocyanin-related genes in shoots of 7-d-old WT and *dps1* seedlings grown on P+ and P- media. The names of the genes examined are indicated on the bottom of each panel. Three independent experiments were performed with similar results and one representative experiment is shown. Values are means ± SD of three technical replicates and represent fold changes normalized to transcript levels of the WT on P+ medium. An asterisk indicates a significant difference relative to the WT (*t*-test, P < 0.05).

**Supplementary Figure 5**


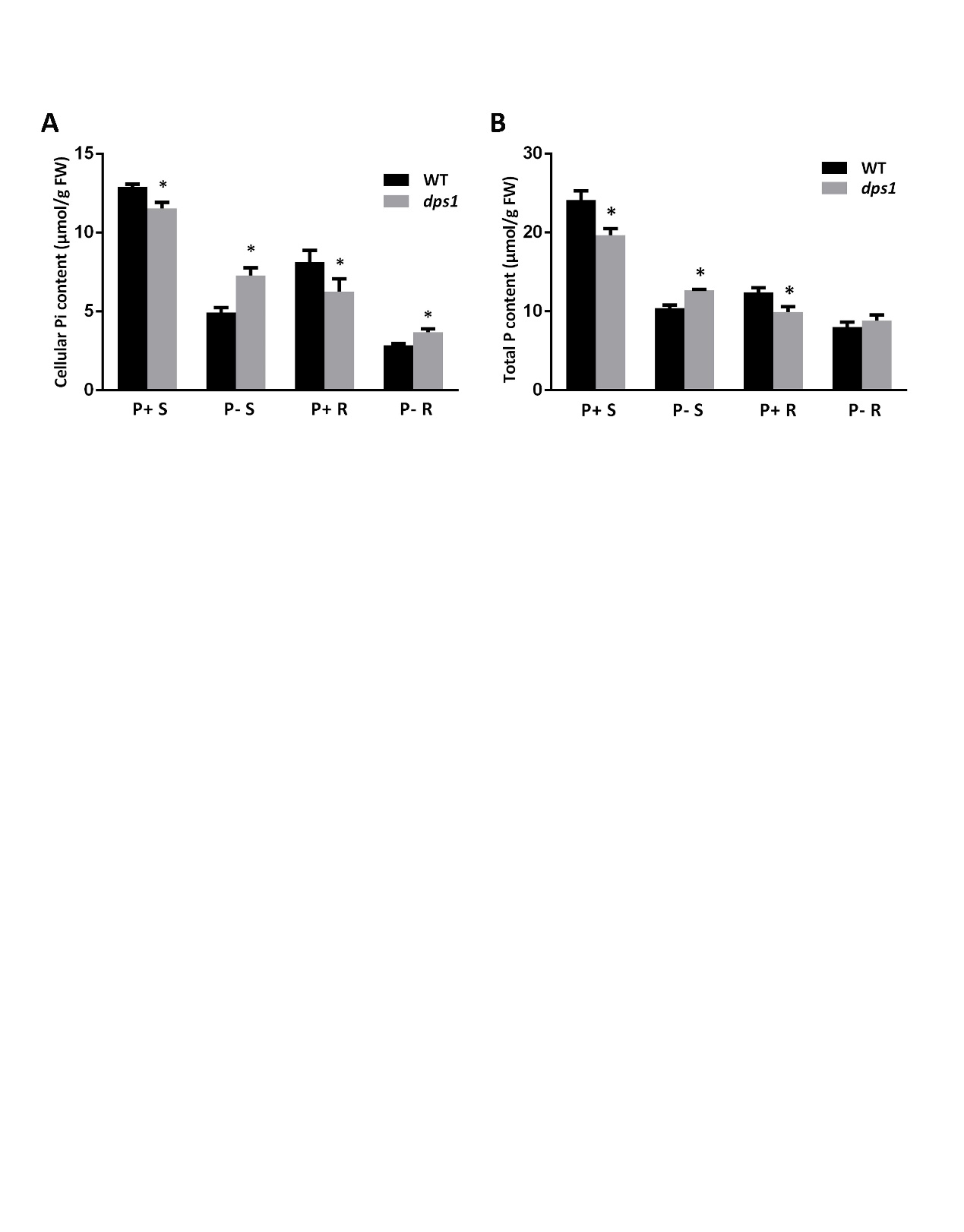


**Supplementary Figure 5.** Cellular Pi and total P contents in WT and *dps1* seedlings. (A) Cellular Pi contents of 7-d-old seedlings grown on P+ or P- media. (B) Total P contents of 7-d-old seedlings grown on P+ or P- media. In (A, B), Three independent experiments were performed with similar results and one representative experiment is shown. Values are means ± SD of three technical replicates. An asterisk indicates a significant difference relative to the WT (*t*-test, P < 0.05). S: shoots, R: roots.

**Supplementary Figure 6**


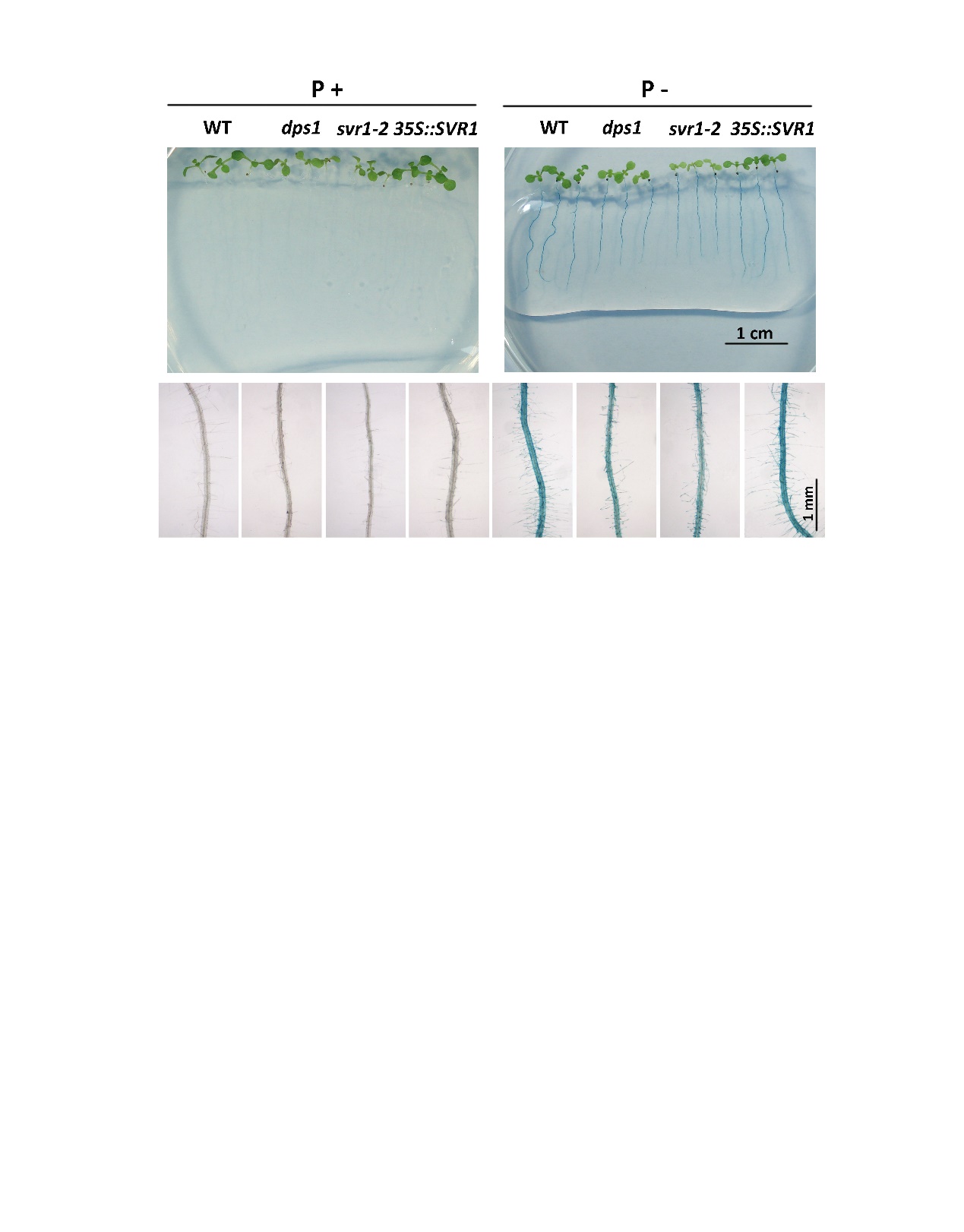


**Supplementary Figure** **6.** Root-associated APase activities of the WT, *dps1*, *svr1-2*, and a complementation line. Top row, APase activities on the root surface of 7-d-old seedlings grown on P+ and P- media as indicated by BCIP staining; bottom row, close views of the BCIP-stained roots in the top row.

**Supplementary Figure 7**


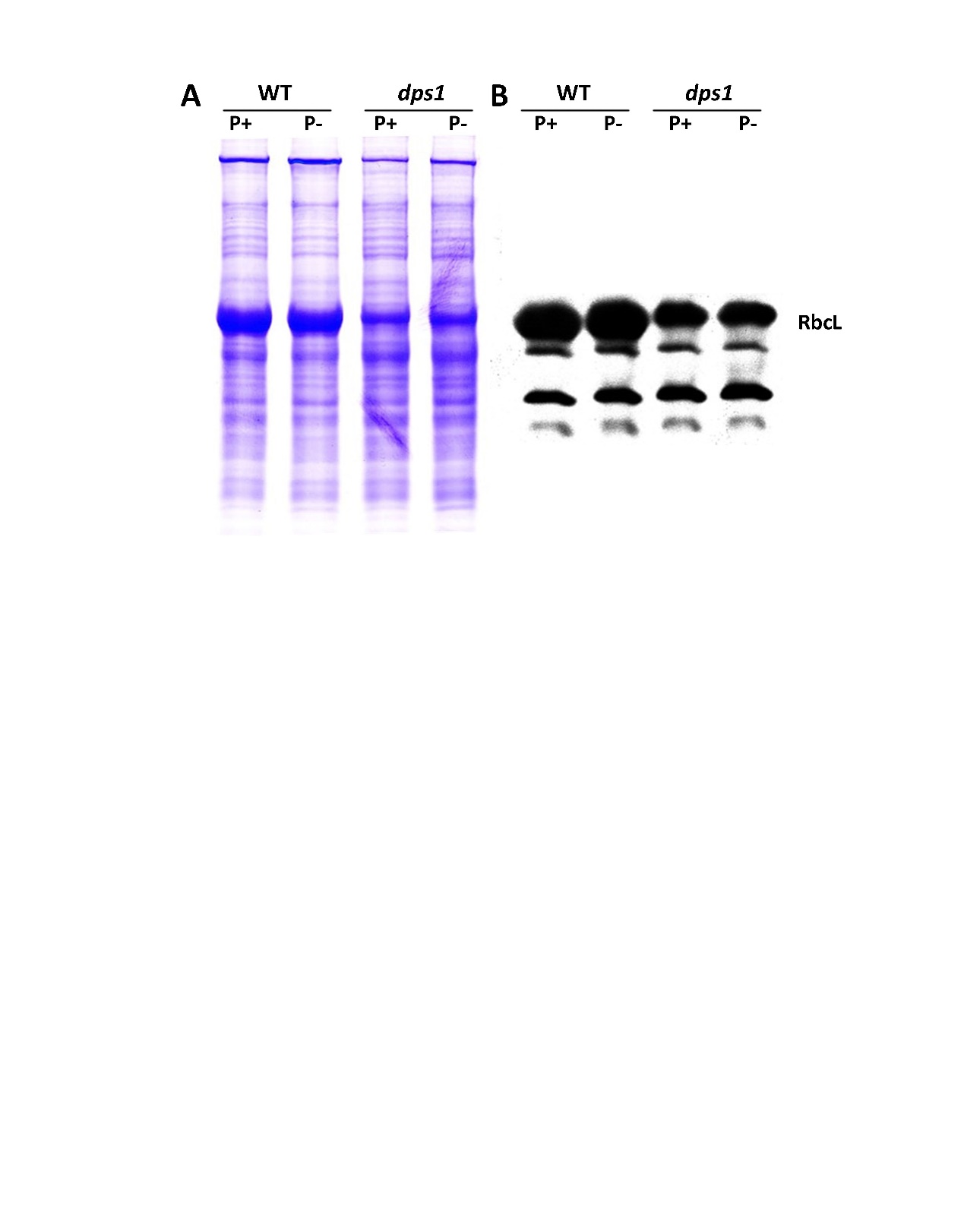


**Supplementary Figure 7.** Accumulation of RbcL proteins in WT and *dps1* seedlings grown on P+ and P- media. (A) Profiles of total proteins extracted from 7-d-old seedlings as indicated by SDS-PAGE analysis. (B) Western blot analysis of the total proteins in (A) using anti-RbcL antibodies.

**Supplementary Figure 8**


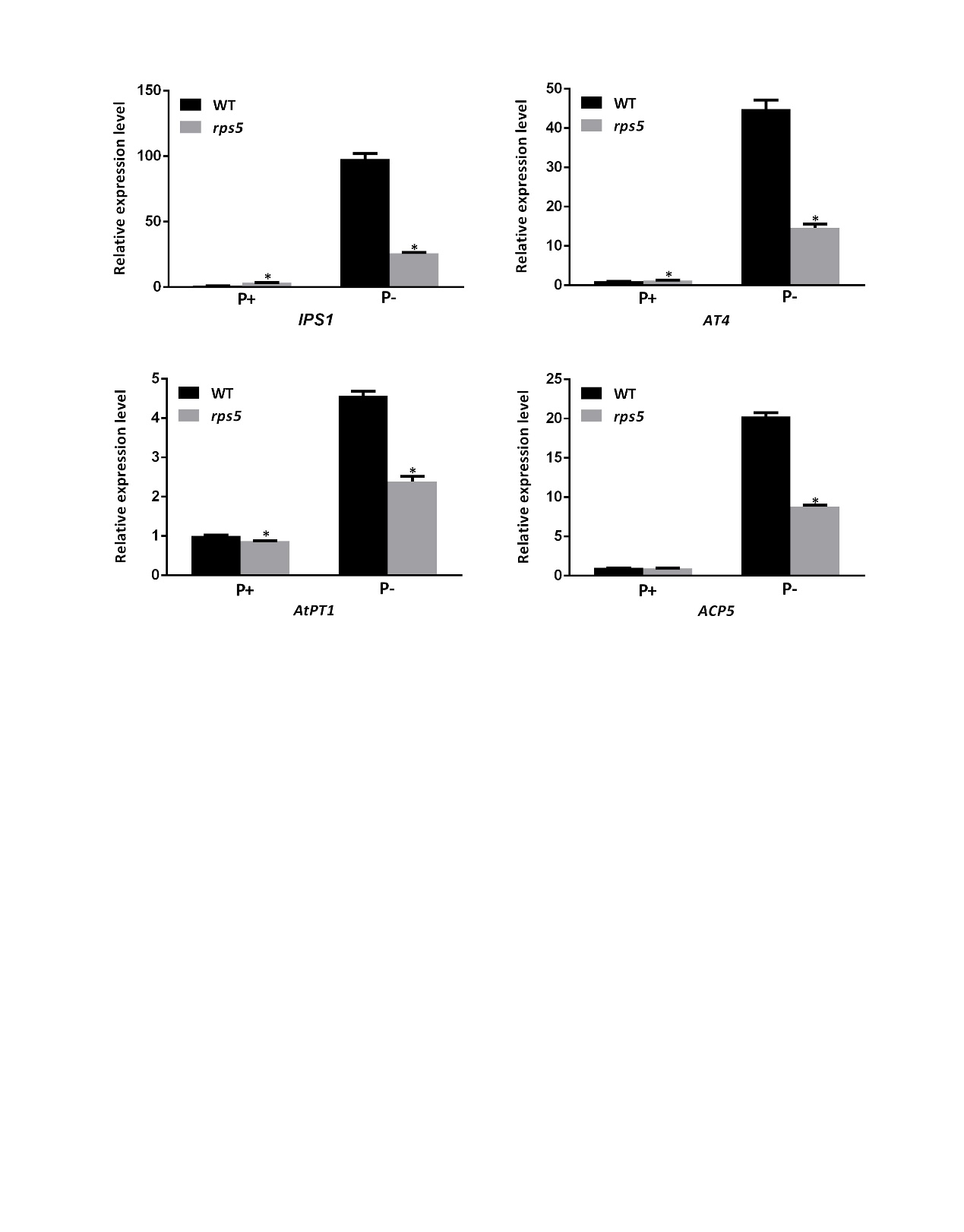


Supplementary Figure 8. Relative expression of four PSI genes in 7-d-old WT and *rps5* seedlings grown on P+ and P- media. The names of the genes examined are indicated on the bottom of each panel. Three independent experiments were performed with similar results and one representative experiment is shown. Values are means ± SD of three technical replicates and represent fold changes normalized to transcript levels of the WT on P+ medium. An asterisk indicates a significant difference relative to the WT (*t*-test, P < 0.05).

**
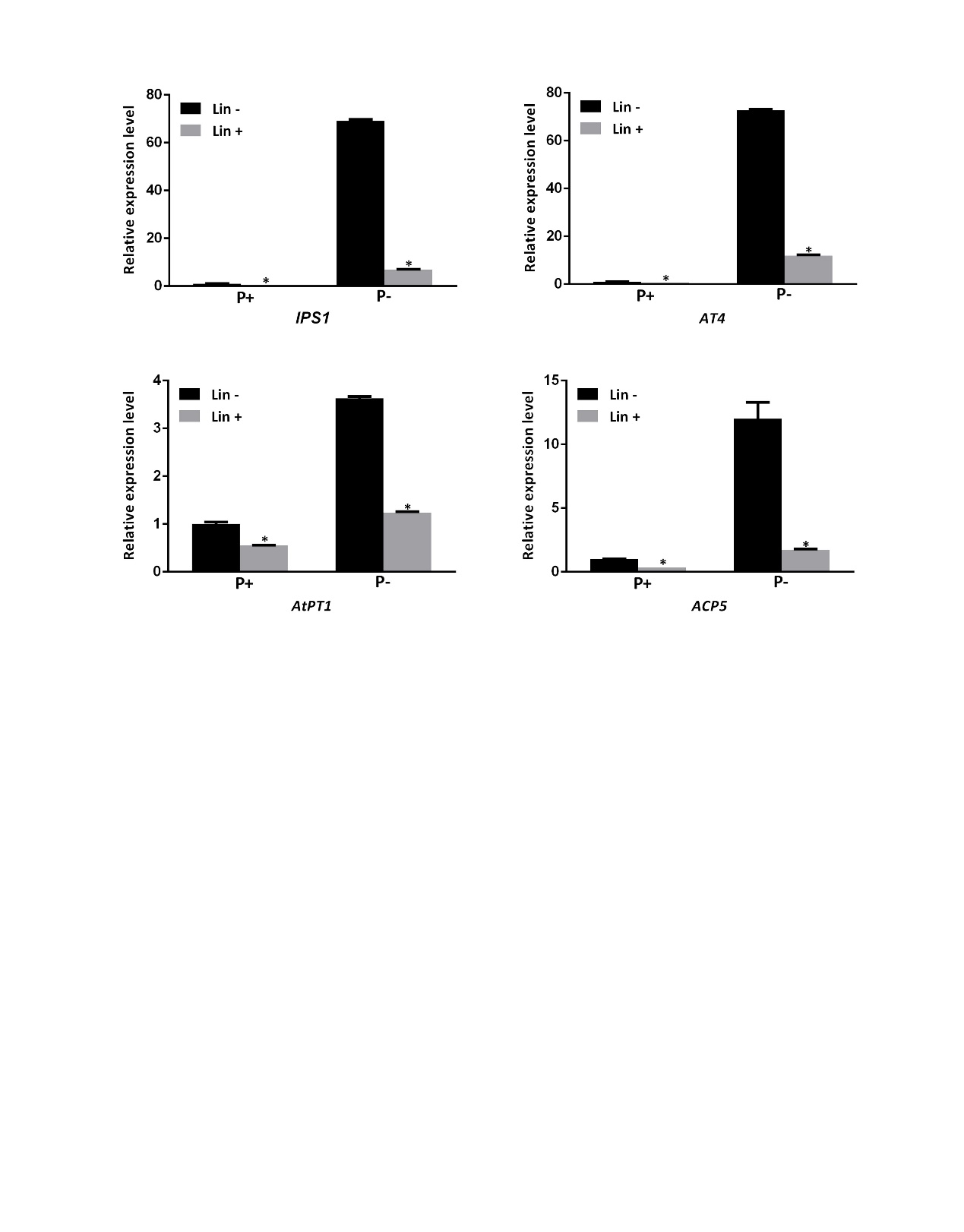
Supplementary Figure 9**

**Supplementary Figure 9.** Relative expression of four PSI genes in 7-d-old WT seedlings grown on P+ and P- media without (Lin-) or with (Lin+) 30 μM lincomycin. The names of the genes examined are indicated on the bottom of each panel. Three independent experiments were performed with similar results and one representative experiment is shown. Values are means ± SD of three technical replicates and represent fold changes normalized to transcript levels of the non-treated WT on P+ medium. An asterisk indicates a significant difference relative to the WT (*t*-test, P < 0.05).
